# A Cytoplasmic Complex Mediates Specific mRNA Recognition and Localization in Yeast

**DOI:** 10.1371/journal.pbio.1000611

**Published:** 2011-04-19

**Authors:** Marisa Müller, Roland Gerhard Heym, Andreas Mayer, Katharina Kramer, Maria Schmid, Patrick Cramer, Henning Urlaub, Ralf-Peter Jansen, Dierk Niessing

**Affiliations:** 1 Institute of Structural Biology, Helmholtz Zentrum München–German Research Center for Environmental Health, München, Germany; 2 Gene Center and Department of Biochemistry, Ludwig-Maximilians-University, München, Germany; 3 Bioanalytical Mass Spectrometry Group, Max Planck Institute for Biophysical Chemistry, Göttingen, Germany; 4 Center for Integrated Protein Science CIPSM, München, Germany; 5 Interfaculty Institute for Biochemistry, University of Tübingen, Tübingen, Germany; Cancer Research UK, United Kingdom

## Abstract

In eukaryotes, hundreds of mRNAs are localized by specialized transport complexes. For localization, transcripts are recognized by RNA-binding proteins and incorporated into motor-containing messenger ribonucleoprotein particles (mRNPs). To date, the molecular assembly of such mRNPs is not well understood and most details on cargo specificity remain unresolved. We used *ASH1*-mRNA transport in yeast to provide a first assessment of where and how localizing mRNAs are specifically recognized and incorporated into mRNPs. By using in vitro–interaction and reconstitution assays, we found that none of the implicated mRNA-binding proteins showed highly specific cargo binding. Instead, we identified the cytoplasmic myosin adapter She3p as additional RNA-binding protein. We further found that only the complex of the RNA-binding proteins She2p and She3p achieves synergistic cargo binding, with an at least 60-fold higher affinity for localizing mRNAs when compared to control RNA. Mutational studies identified a C-terminal RNA-binding fragment of She3p to be important for synergistic RNA binding with She2p. The observed cargo specificity of the ternary complex is considerably higher than previously reported for localizing mRNAs. It suggests that RNA binding for mRNP localization generally exhibits higher selectivity than inferred from previous in vitro data. This conclusion is fully consistent with a large body of in vivo evidence from different organisms. Since the ternary yeast complex only assembles in the cytoplasm, specific mRNA recognition might be limited to the very last steps of mRNP assembly. Remarkably, the mRNA itself triggers the assembly of mature, motor-containing complexes. Our reconstitution of a major portion of the mRNA-transport complex offers new and unexpected insights into the molecular assembly of specific, localization-competent mRNPs and provides an important step forward in our mechanistic understanding of mRNA localization in general.

## Abstract 2

The localization of ash mRNA in yeast requires the binding of She2p and the myosin adaptor protein She3p to its localization element, which is highly specific and leads to the assembly of stable transport complexes.

## Author Summary

In eukaryotes, the majority of cells are asymmetric and a way to establish such polarity is directional transport of macromolecules along cytoskeletal filaments. Among the cargoes transported, mRNAs play an essential role, as their localized translation contributes significantly to the generation of asymmetry. To date, hundreds of asymmetrically localized mRNAs in various organisms have been identified. These mRNAs are recognized by RNA-binding proteins and incorporated into large motor-containing messenger ribonucleoprotein particles (mRNPs) whose molecular assembly is poorly understood. In this study, we used the well-characterized process of *ASH1*-mRNA transport in *Saccharomyces cerevisiae* to address the question of how localizing mRNAs are recognized and specifically incorporated into mRNPs. Surprisingly, we found that the previously implicated mRNA-binding proteins She2p and Puf6p do not bind to cargo mRNAs with high specificity. Instead, the cytoplasmic motor-adapter protein She3p is responsible for synergistic cargo binding with She2p and for the stable incorporation of specific localizing mRNA into the transport complex. We propose that the specific recognition of localizing mRNAs happens at the very last step of cytoplasmic mRNP maturation. Other organisms might employ similar mechanisms to establish cellular polarity.

## Introduction

In eukaryotes, directional transport and localization of mRNA is widely used to regulate gene expression on a temporal and spatial level. mRNA localization is involved in diverse processes such as inducing cellular asymmetry, guiding key events during embryonic development, and supporting synaptic plasticity [1]–[4]. For these processes, motor-containing mRNPs usually translocate translationally silent transcripts from perinuclear areas to their subcellular destination. After anchoring, mRNA translation is activated and encoded proteins are produced [2]–[4]. To date, the basic principles underlying the incorporation of mRNAs into translocating particles, the specific roles of mRNP-core factors, and subsequent mRNA localization are not well understood. Thus, a detailed analysis is required on how mRNA-translocation particles assemble and how RNA-cargo specificity is achieved.

A study on the localization of *Vg1* and *VegT* RNPs in *Xenopus* oocytes already showed marked differences between the nuclear and cytoplasmic protein composition of these RNPs [5]. By using immunoprecipitation experiments on cell extracts and antibody stainings, the authors demonstrated that factors sequentially join the maturing complex on its path from the oocyte nucleus to the cytoplasmic vegetal pole. Comparably little biochemical data are available explaining how mRNPs recognize their cargo-transcripts in a specific way. Available studies only revealed a 3–7-fold higher affinity for localizing mRNAs when compared to non-localizing RNAs [6],[7]. Because such rather small differences are unlikely to explain the highly selective transport of RNAs observed in vivo, it seems obvious that essential information on the assembly of specific transport complexes is missing. The goal of the present work was to understand such general principles by in vitro reconstituting a major part of a core mRNA-transport complex from yeast and complementing in vivo studies.

During mitosis of *Saccharomyces cerevisiae*, *ASH1* mRNA is transported as part of large mRNPs from the mother cell to the daughter cell [4],[8]. *ASH1* mRNA contains four *cis*-acting regions, termed zip-code elements E1, E2A, E2B, and E3, mediating mRNA incorporation into the mRNPs and its subsequent localization [9],[10]. After mRNP anchoring, *ASH1* mRNA is translated in the daughter cell. The protein product Ash1p acts as a repressor of mating-type switching exclusively in the daughter cell [11],[12]. In addition to *ASH1* mRNA, more than 30 transcripts are localized by this transport complex [13]–[15]. Their incorporation into the mRNP is thought to be mediated by its core RNA-binding protein She2p [16]–[18].

She2p is an unusual RNA-binding protein [19]. It interacts with *ASH1* mRNA already in the nucleus at the site of transcription [20] and escorts it into the cytoplasm [7],[16],[21]. After nuclear export, the She2p-*ASH1* mRNA complex binds to the stable cytoplasmic co-complex of the myosin-adapter She3p and the type V myosin motor Myo4p [17],[18],[22]–[26]. Recent actin-gliding assays with mRNPs purified from yeast extracts showed that a core complex consisting of Myo4p, She3p, She2p, and a shortened *ASH1*-E3 RNA element has motile activity [27].

In addition to this cytoplasmic core mRNP, the RNA-binding proteins Puf6p and Khd1p associate with the *ASH1* mRNA-She2p complex and are required for efficient *ASH1*-mRNA localization in vivo [28]–[30]. Whereas the nucleo-cytoplasmic shuttling protein Puf6p binds to *ASH1*-E3, cytoplasmic Khd1p interacts with a region encompassing *ASH1*-E1 RNA. Both proteins are involved in translational repression during transport [28]–[30].

Among all these factors, She2p is the only RNA-binding protein known to bind to all four zip-code elements of the *ASH1* mRNA [9],[10],[17],[31] and to be part of the core-transport complex [27]. She2p is therefore believed to mediate the specific incorporation of localizing mRNAs into the *ASH1* mRNP. To date, it is unclear how She2p mediates this function.

Since the *ASH1*-E3 zip code alone can mediate efficient mRNA localization in vivo [27],[31],[32], we concentrated our reconstitution studies on this element. Because Khd1p does not interact with the E3 element [32], we excluded this protein from our study.

We found that She2p and Puf6p bind to zip-code elements only with moderately higher affinity than to unrelated RNAs. This raised the question of when and how specificity for mRNP transport is achieved. We further discovered that the myosin adapter She3p is a previously uncharacterized RNA-binding protein. It interacts directly with She2p also in absence of RNA. Addition of zip code containing RNAs results in the formation of highly specific ternary complexes. This specific mRNP has an at least 60-fold higher affinity for zip-code RNAs. Furthermore, incorporation of the RNA increases the interaction between She2p and She3p to a similar extent. Since we find She3p exclusively in the cytoplasm, we propose that highly specific mRNA recognition occurs after nuclear export and is coupled to the assembly of mature, motor-containing transport complexes.

## Results

### She2p Binds to Unrelated Stem-Loop RNAs

**Figure 1:**
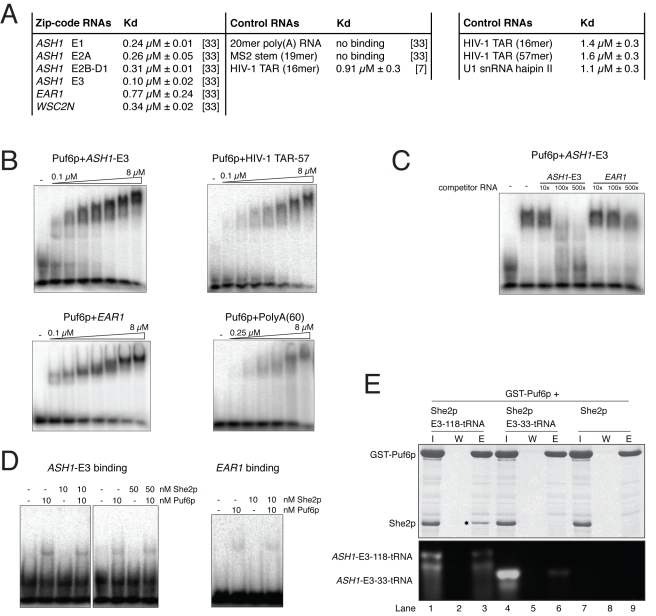
Interaction studies with Puf6p, She2p, and RNA. (A) RNA-binding studies with She2p. Left table summarizes previously published results of RNA filter-binding assays. Numbers in parentheses indicate respective references. Right table summarizes filter-binding assays with control RNAs performed in this study. The data indicate that She2p binds to localizing RNAs and control RNAs with only a modest difference in Kd. (B) EMSAs show binding of Puf6p to the *ASH1*-E3 zip code comprising two *PUF*-consensus sites, the *PUF*-consensus site-lacking *EAR1* zip code, the HIV-1 TAR stem-loop, and a 60 mer poly-A RNA. (C) Competitive EMSA with Puf6p and *ASH1*-E3 zip-code RNA reveals a preference of Puf6p for the *ASH1*-E3 zip code. Puf6p and *ASH1*-E3 RNA were incubated with 10-fold, 100-fold, or 500-fold excess of unlabeled *ASH1*-E3 RNA or *EAR1* RNA. (D) Puf6p and She2p do not bind synergistically to *ASH1*-E3 RNA. In EMSAs with *ASH1*-E3 RNA, Puf6p, and She2p, no increase in affinity was observed. Likewise, using the *EAR1* zip code as control, no difference in affinity was detected. Please note that She2p alone interacts with RNA too transiently for detection in EMSAs. (E) Pull-down experiments with immobilized GST-Puf6p, She2p, and *ASH1*-E3 RNA fused to tRNA show an RNA-dependent interaction of Puf6p and She2p (lanes 1–3; asterisk highlights bound She2p). No interaction was observed when a control RNA (*ASH1* E3-33-tRNA fusion) was used (lanes 4–6) or when RNA was omitted from the reaction (lanes 7–9) (I, input; W, final wash; E, elution). Upper part shows SDS-PAGE; lower part shows an agarose gel.

Previous studies showed that She2p (Gene ID: 853728) binds to all four zip-code elements of the *ASH1* mRNA (Gene ID: 853650) [9],[10]. It also binds to the zip-code element of *EAR1* mRNA (Gene ID: 855207) [13] and to the 5′ zip-code element (*WSC2N*) of *WSC2* mRNA (Gene ID: 855438) [13]. The equilibrium-dissociation constants (Kd) of She2p binding to these elements are all in the nanomolar range (Figure 1A, left table) [33]. The strongest binding with a Kd of 0.10 µM was observed for the most 3′-located E3 element of the *ASH1* mRNA and the weakest binding for the *EAR1* zip-code element (Kd = 0.77 µM). No binding was detected to an unstructured Poly-A_20_ RNA or the MS2-stem-loop RNA [33]. However, we had previously also observed considerable She2p binding to the stem-loop containing RNA HIV-1 TAR (16 mer) (Kd = 0.91 µM; Figure 1A, left table) [7]. We therefore wondered whether this binding to an unrelated RNA is an exception or constitutes a more general feature of She2p.

When repeating RNA filter-binding experiments with HIV-1 TAR RNA (16 mer), a longer HIV-1 TAR RNA (57 mer), and the U1 snRNA hairpin II as controls, we observed Kds ranging from 1.1 µM to 1.6 µM (Figure 1A, right table). These data indicate that the affinity of She2p to zip-code elements is only about 2–10-fold higher than to unrelated stem-loops. In vivo, however, only few mRNAs are associated with She2p [13]–[15]. Thus, the low in vitro specificity of She2p binding is unlikely to explain the high selectivity for localizing mRNAs in the cell.

One possible explanation for the high specificity in vivo is that another, more selective RNA-binding protein mediates specific binding to localizing transcripts. A recent study suggested that Puf6p (Gene ID: 852107), which binds to the *ASH1-E3* zip-code element, also interacts directly with She2p in vivo [21]. Thus, Puf6p could potentially bind specifically to *ASH1* mRNA and simultaneously to She2p, mediating cargo specificity for the transport complex.

### Puf6p Interacts Indirectly through RNA-Binding with She2p and Shows Limited RNA Specificity

We purified Puf6p to near homogeneity (see Materials and Methods) and confirmed its integrity by size-exclusion chromatography and circular dichroism spectroscopy (Figure S1). Then we assessed the specificity of Puf6p binding to RNAs by electrophoretic mobility shift assays (EMSA) and observed a rather indiscriminate binding of Puf6p to RNAs (Figure 1B). Also the concentration-dependent increasing size of the shifted RNA complexes hints at an unspecific binding of additional Puf6p molecules to RNA. We repeated these experiments with *ASH1*-E3 RNA in the presence of excess amounts of either specific (*ASH1* E3) or unspecific (*EAR1*) unlabeled competitor RNA. The *EAR1* zip-code element was chosen as an unspecific competitor because it lacks a *PUF*-consensus site [29]. Complex formation of *ASH1*-E3 RNA and recombinant Puf6p was competed for by excess amounts of *ASH1*-E3 RNA, but only poorly by *EAR1* RNA (Figure 1C). Thus, in vitro Puf6p displays a limited preference for RNAs with *PUF*-consensus sites.

In order to find out if the combination of She2p and Puf6p results in synergistic RNA binding, we also performed EMSAs with both proteins and RNAs. In none of these experiments did we observe any synergistic binding or increased specificity (Figure 1D).

To assess the previously suggested direct interaction of Puf6p with She2p in vitro [21], we performed pull-down experiments with the respective recombinant proteins. Using GST-Puf6p and She2p, no stable interaction was observed in absence of RNA (Figure 1E). Next, we investigated whether Puf6p and She2p interact in an RNA-dependent manner. In order to obtain the large amounts of RNA required for these experiments, we used a recently established expression strategy, in which RNAs of interest are recombinantly expressed in fusion with tRNA [34],[35]. Wild-type *ASH1*-E3 RNA and a shortened control *ASH1*-E3 RNA, which fails to bind She2p (unpublished data) and lacks predicted Puf6p binding sites [29], were expressed and purified in fusion to the anticodon stem of a methionine tRNA (E3-118-tRNA and E3-33-tRNA, respectively; Figure S2). When adding E3-118-tRNA, we observed both Puf6p and She2p in the same complex, whereas no interaction was detected with the control E3-33-tRNA (Figure 1E). Thus, the interaction between Puf6p and She2p observed in vitro appears to be indirect. Furthermore, from the sum of these experiments we can conclude that Puf6p is unlikely to mediate cargo-specificity to the transport complex.

### She2p and She3p Interact Directly with Each Other

**Figure 2:**
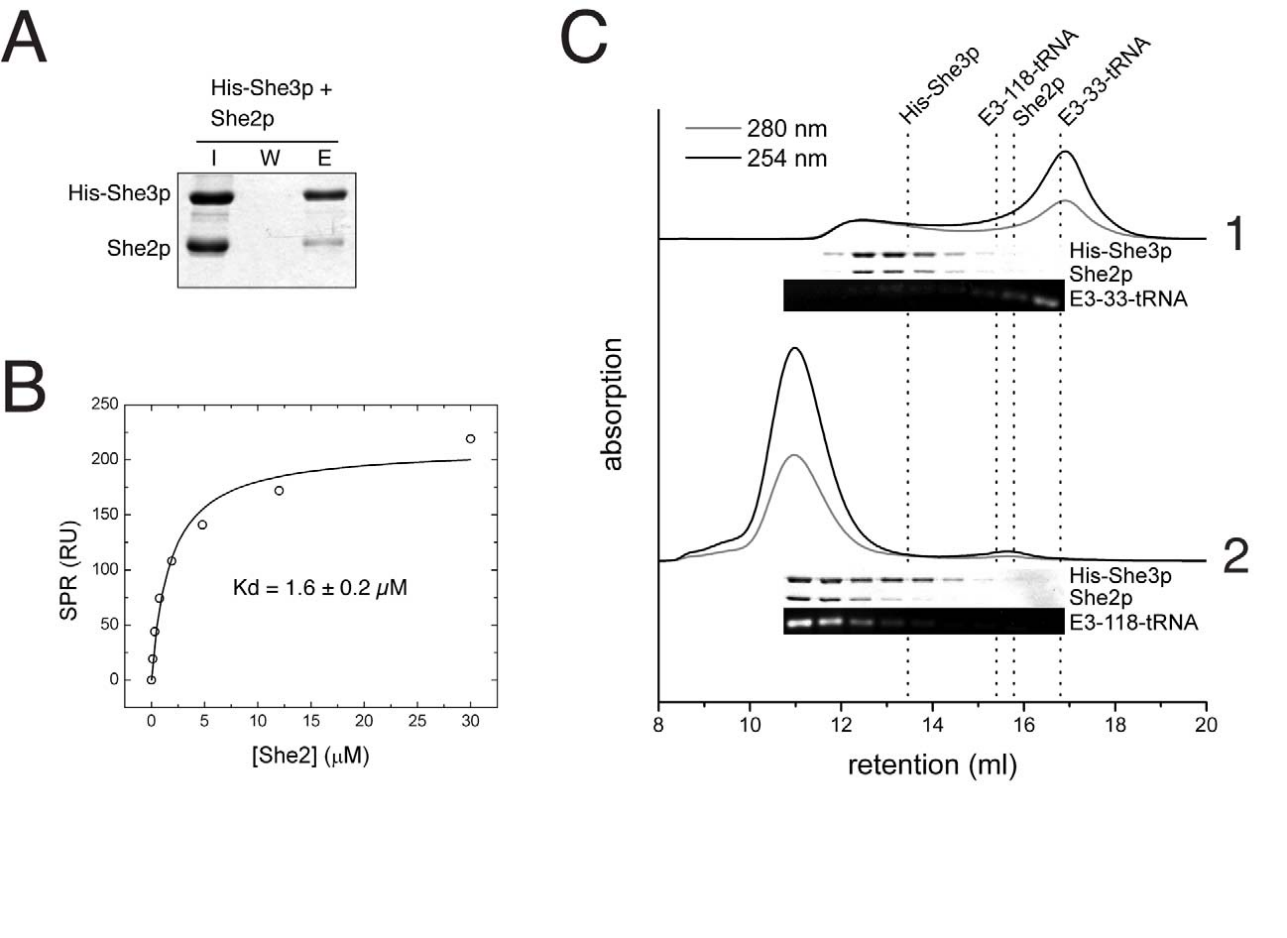
She2p and She3p interact directly and form a ternary complex with RNA. (A) In pull-down experiments with nickel sepharose, She2p co-purified with His-tagged She3p (I, input; W, final wash; E, elution). (B) In steady-state surface plasmon resonance experiments, She2p bound to surface-coupled His-She3p with a Kd of 1.6±0.2 µM (mean and deviation of two independent experiments). (C) In analytical size-exclusion chromatography, She2p and His-She3p eluted as a ternary complex with *ASH1* E3-118-tRNA (chromatogram 2) but not with the non-functional *ASH1* E3-33-tRNA (chromatogram 1). Corresponding fractions were analyzed by SDS-PAGE and agarose gel electrophoresis and are shown below each chromatogram. Dotted lines indicate the peak retention volumes of the individual components.

In the cytoplasm, the cargo adapter She3p (Gene ID: 852427) joins the She2p:RNA complex. She3p is thought to connect specifically pre-bound mRNAs to the motor Myo4p (Gene ID: 851204) [17],[18],[22]–[26]. However, the weak RNA-binding specificities we observed for She2p and Puf6p appear inconsistent with this model. Therefore, we assessed the role of She3p in complex formation. In pull-down experiments with His-tagged She3p and in size-exclusion chromatography, we observed a co-purification of She2p (Figures 2A and S3A,B). Subsequent surface plasmon resonance (SPR) experiments with amine-coupled She3p yielded a Kd of 1.6±0.2 µM (Figure 2B) and fast complex dissociation (unpublished data), suggesting low complex stability.

### She2p, She3p, and Zip-Code RNAs Form Stable Ternary Complexes

Since previous data indicated that She3p might be able to join the co-complex of She2p and RNA [17], we also tested their assembly with recombinant proteins by size-exclusion chromatography. She2p and She3p indeed co-eluted efficiently with zip code containing E3-118-tRNA at high molecular weight (Figure 2C). In contrast, the control E3-33-tRNA did not elute in co-complex with both proteins. Reconstitution could be further expanded by the addition of the C-terminal half of Myo4p (Figure S3C), which interacts with She3p. In summary, these observations show that She2p, She3p, and zip-code RNA form a ternary complex that can be reconstituted with recombinant proteins in vitro. More importantly, it suggests that only functional zip-code RNAs join the She2p:She3p complex.

### Puf6p Is Part of the Cytoplasmic Complex with She2p, She3p, and *ASH1*-E3 RNA

Because Puf6p co-purifies with She2p and *ASH1* mRNA and colocalizes with *ASH1* mRNA at the bud tip, it is considered to be associated with the actively transported *ASH1* mRNPs [28],[29]. Thus, Puf6p should also associate with the recombinant She2p:She3p:E3-RNA complex. We performed in vitro pull-down experiments with either His-tagged She3p or GST-tagged Puf6p and analyzed the co-elution with other complex factors. Using either strategy, Puf6p co-purified with She2p, the zip-code element-containing E3-118-tRNA, and She3p (Figure S4A–C). In contrast, in absence of RNA or when the *PUF* consensus-lacking E3-33-tRNA was used, Puf6p did not form a detectable complex with She2p and She3p (Figure S4A,B). These results indicate that Puf6p and the She2p:She3p co-complex can indeed bind simultaneously to the *ASH1*-E3 element. It supports the previously reported role of Puf6p as translational repressor of the actively transporting *ASH1* mRNP [28],[29].

### She3p Is an RNA-Binding Protein

**Figure 3:**
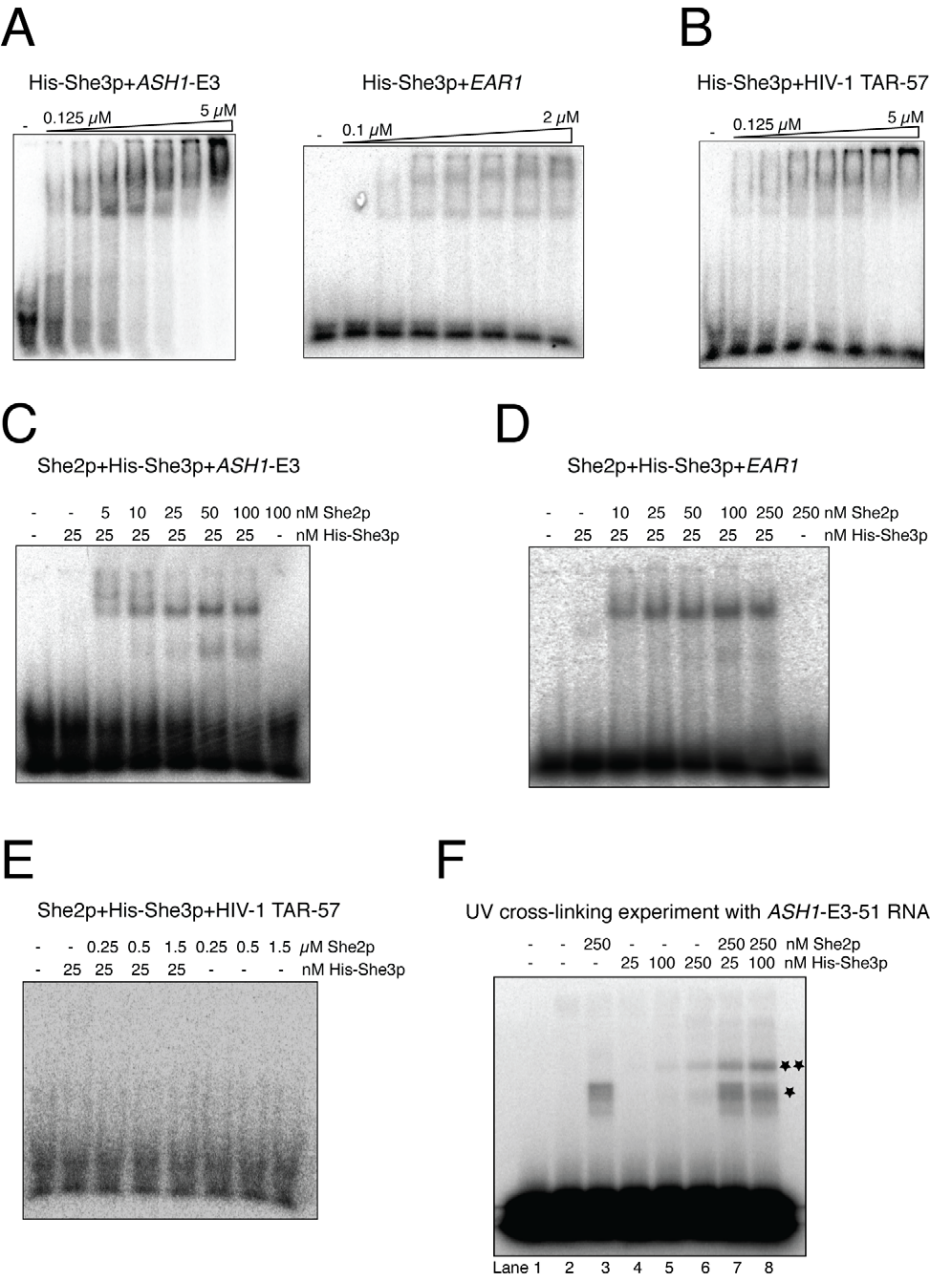
RNA binding by She3p and by its co-complex with She2p. (A) She3p is an RNA-binding protein. EMSAs reveal strong binding of She3p to zip-code elements of *ASH1* and *EAR1* as well as (B) to the HIV-1 TAR stem-loop. (C–E) She2p and She3p form specific ternary complexes with zip-code RNAs. EMSAs with a constant She3p concentration (25 nM) and varying amounts of She2p show strong complex formation with the *ASH1*-E3 zip code (C) and the *EAR1* zip code (D). The Kd for these specific complexes was estimated to be around 25 nM. In absence of either protein, no stable RNA-protein complexes were observed. (E) With the HIV-1 TAR RNA, no complexes could be detected even at 1.5 µM She2p concentration. (F) UV cross-linking followed by denaturing PAGE proved direct *ASH1*-E3 RNA binding by She2p and She3p in the ternary complex. The radioactively labeled minimal *ASH1*-E3 element of 51 bases (Figure S2) efficiently cross-linked with She2p alone (lane 3), She3p alone (lanes 5–6), and with both proteins in the ternary complex (lanes 7–8). Lane 1 shows RNA without and lane 2 after UV treatment. One asterisk marks the cross-linking band of She2p; two asterisks mark She3p cross-links.

To understand the specific role of She3p in ternary complex assembly, we also tested if She3p alone binds to RNA. In EMSAs She3p indeed bound to zip-code RNAs (Figure 3A) but also to HIV-I TAR RNA (Figure 3B) with affinities comparable to She2p [7],[33]. Thus, we consider She3p to be a previously undetected, rather unspecific RNA-binding protein. Remarkably, it fails to show sequence similarity to known RNA-binding domains. As with other RNA-binding proteins tested in this study, She3p alone lacks RNA-binding specificity suitable to explain the highly specific transport of a subset of mRNAs in vivo.

### She2p and She3p Act Synergistically to Form RNA Complexes with High Affinity and Specificity

Next, we assessed whether the cytoplasmic She2p:She3p complex shows higher specificity for zip-code RNAs than the individual proteins. Whereas the interaction of She2p with zip-code RNAs was barely detectable by EMSAs (Figure S5A,B), She3p binding was observed even at low nanomolar concentrations (Figure S5C). In order to detect potential synergisms in binding, we performed EMSAs with increasing concentrations of She2p and a constant, low concentration of She3p (25 nM). At these concentrations, She2p alone did not show any mobility shift (Figure S5A,B), whereas She3p yielded a faint shift for the *ASH1*-E3 element (Figure S5C). In the event of synergistic RNA binding by both proteins, a distinct and stronger band shift corresponding to the ternary complex would be expected.

EMSAs with She2p, She3p, and *ASH1*-E3 RNA showed indeed strong binding even at the lowest experimental She2p concentration of 5 nM (Figures 3C and S5D). Subsequent Western blot analysis confirmed that She2p is present in the detected RNA-protein complexes (Figure S5E). A similar increase in affinity was also observed with the *EAR1* zip-code element (Figure 3D) and the *ASH1*-E1, E2A, and E2B zip-code elements (Figure S5F–H). Based on these EMSAs, we estimated the Kds for ternary complex formation with all four *ASH1* zip-code elements and the *EAR1* zip-code element to be around 25 nM. When repeating these EMSAs with unspecific HIV-1 TAR RNA, no complexes were observed even at experimental She2p concentrations of 1.5 µM (Figure 3E). Thus, it can be estimated that the She2p:She3p co-complex assembles with all tested zip code containing RNAs with at least 60-fold higher affinity. It should be noted that these numbers only provide a rough estimate. They nevertheless clearly indicate that this complex is highly specific.

In order to confirm the synergistic effect observed in EMSAs by a different approach, we also performed filter-binding assays. These experiments with the *ASH1*-E3 and *EAR1* zip-code elements also yielded a clear synergistic effect whenever combinations of She2p and She3p were added (Figure S6A,B).

### She2p and She3p Directly Bind to RNA in the Ternary Complex

Although She2p and She3p are both RNA-binding proteins, it is possible that in the ternary complex only one of them directly contacts the RNA. In such a scenario the effect of the other protein would be limited to an allosteric influence on the RNA-interacting protein. In order to test this possibility, we performed UV cross-linking experiments with She2p, She3p, and radioactively labeled *ASH1*-E3 RNA. In order to optimize the experimental conditions, we employed a shortened E3 RNA of 51 nucleotides (Figure S2) that still mediated synergistic RNA binding with She2p and She3p (Figure S5I). Following UV cross-linking and denaturing gel electrophoresis, we observed distinct bands for She2p (Figure 3F, lane 3) and for She3p (Figure 3F, lanes 5–6). Those bands for She2p and She3p were both detected also when the ternary complex was subjected to UV cross-linking (Figure 3F, lanes 7–8). Since UV light only cross-links direct atomic interactions between proteins and nucleic acids, this experiment indicates a direct RNA binding of She2p and She3p within the ternary complex.

### The C-Terminus of She3p Mediates Synergistic RNA Binding with She2p

**Figure 4:**
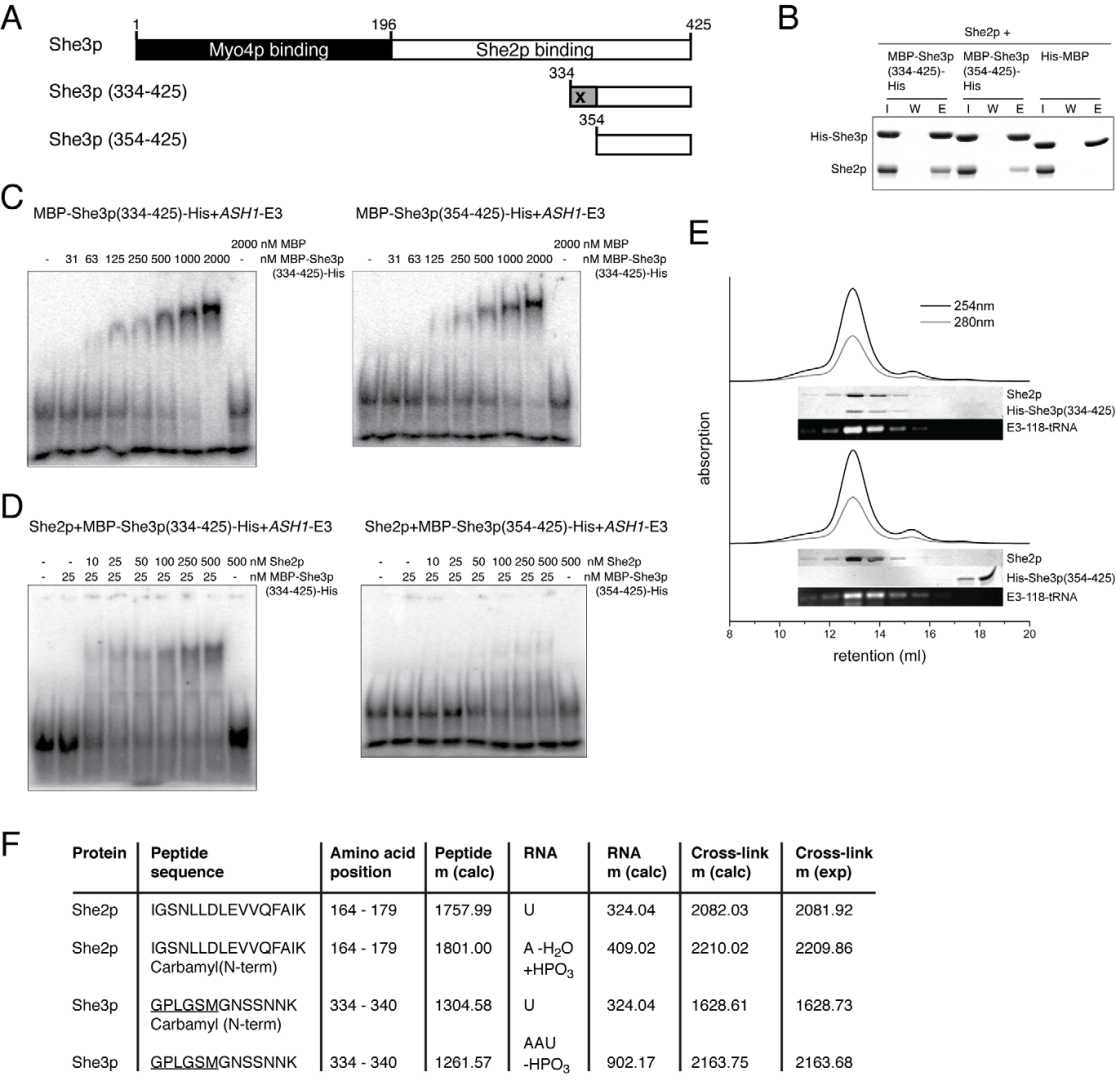
A C-terminal fragment of She3p mediates synergistic interaction with She2p and RNA. (A) Schematic drawing of C-terminal fragments tested in this study. “X” marks the location of the UV cross-linked peptide indicated in (F). (B) In pull-down assays with nickel sepharose, MBP-She3p(334–425)-His efficiently bound to She2p, whereas MBP-She3p(354–425)-His showed a somewhat weaker She2p interaction. (C) Both C-terminal She3p fragments bind efficiently to *ASH1*-E3 RNA. However, RNA binding of MBP-She3p(354–425)-His was slightly weaker than of the longer MBP-She3p(334–425)-His fragment. (D) In EMSAs, only the longer MBP-She3p(334–425)-His supports synergistic RNA binding with She2p. (E) Also in size-exclusion chromatography, only the longer His-She3p (334–425) fragment supports stable ternary complex formation. Please note that the C-terminal She3p constructs do not show any absorption at 280 nm or 254 nm. (F) In the ternary complex consisting of She2p, His-She3p (334–425), and *ASH1*-E3-51 RNA both proteins directly interact with the RNA. After UV cross-linking of the ternary complex, an RNase treatment, proteolytic cleavage, and TiO_2_ enrichment were applied. Peptide-RNA cross-links were subsequently identified by mass spectrometry. The underlined amino acids represent a linker sequence that is not part of She3p.

She3p does not share sequence homology to known protein structures. In order to identify features in She3p that mediate RNA binding, She2p interaction, and ternary complex formation, we analyzed different deletion fragments of She3p. We noticed that a fragment consisting of the very C-terminal 72 amino acids, termed She3p (354–425) (Figure 4A), could be expressed in *Escherichia coli* as a stable protein. This fragment bound RNA and She2p but failed to mediate synergistic formation of the ternary complex (Figure 4B–E and Table 1). A slightly longer fragment of 92 amino acids (Figure 4A), termed She3p (334–425), showed slightly stronger binary interactions with She2p and RNA (Figure 4B,C and Table 1). In contrast to the shorter C-terminal fragment, She3p (334–425) also supported synergistic RNA binding (Figure 4D,E and Table 1). Interestingly, these additional 20 amino acids in the longer C-terminal She3p fragment harbor residues previously described to be important for *ASH1*-mRNA localization in vivo [36].

**Table 1:** Summary of the defects observed upon mutations in She2p or She3p.

Protein	Synergistic RNA Binding	RNA Binding	She2p/She3p Interaction
**She3p (wt)**	+++	+++	+++
She3p (334–425)	+++	+++	++ (*)
She3p (354–425)	+	++	+
She3p (R341E)	+++	n.d.	n.d.
She3p (S343E S361E) [36]	+++	+++	+++
She3p (S348E) [36]	++	+++	+++
She3p (L364A V367A)	+	+++	—
**She2p (wt)**	+++	+++	+++
She2p (ΔhE)	—	+	—
She2p (ΔC)	++	+	+++
She2p (E183A D184A G185A)	+++	n.d.	n.d.
She2p (T191A D192A)	+++	n.d.	n.d.
She2p (F195A L196A)	+	++	—
She2p (Q197A E198A I199A)	+	++	—

“Synergistic RNA binding” was assessed by EMSAs, She2p “RNA binding” by filter-binding assays, She3p “RNA binding” by EMSAs, and “She2p/She3p interaction” by pull-down experiments. All RNA binding experiments were performed with *ASH1*-E3-118 RNA. “+++” indicates wt-like, “++” moderately reduced, “+” reduced, and “—” abrogated binding. “n.d.” indicates that this experiment was not performed and (*) marks that the binding was wt-like in pull-down but reduced in size-exclusion chromatography experiments.

### UV Cross-Linking of the Ternary Complex Followed by Mass-Spectrometric Analysis

**Figure 5:**
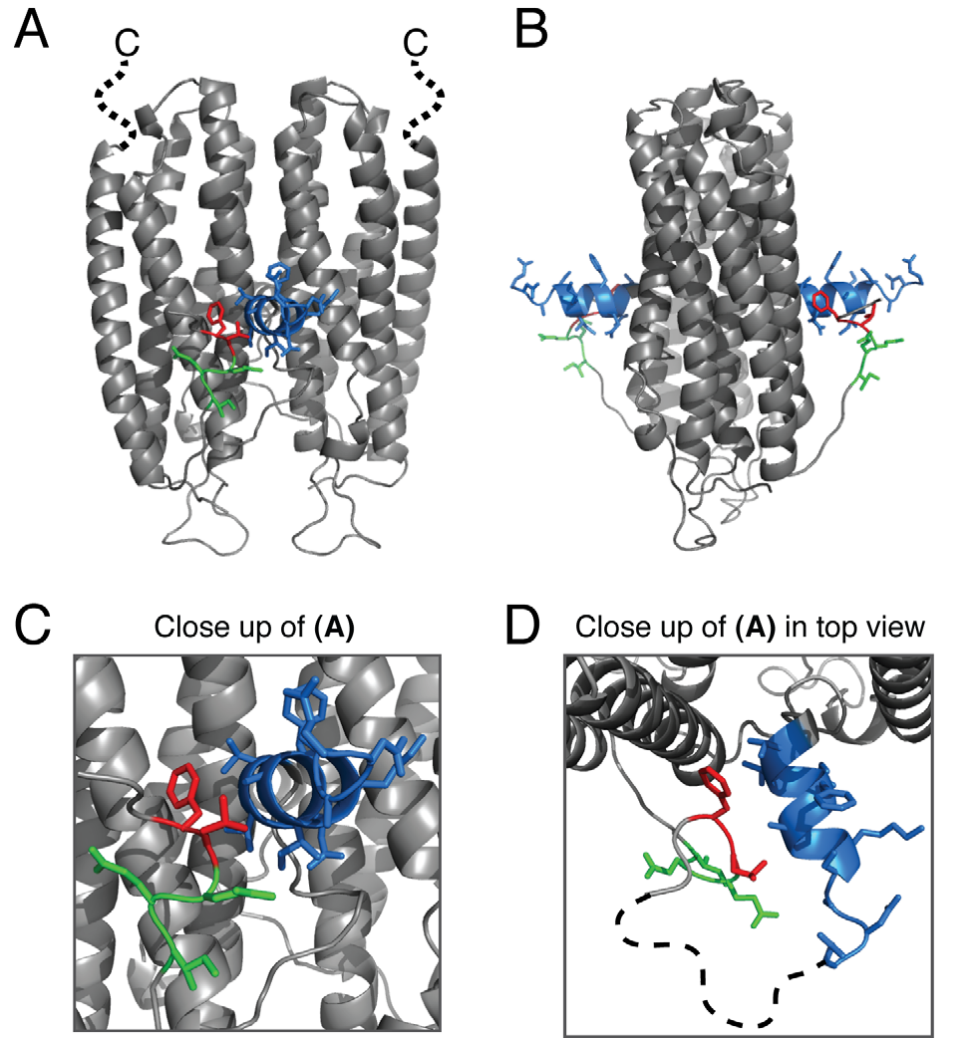
Mutational studies on She2p affecting synergistic RNA binding with She3p. (A) Cartoon representation of the dimeric She2p crystal structure (PBD ID: 1XLY) in front view. The highlighted features were mutated in this study. For simplicity reasons, the positions of helix E and the C-terminus are depicted only on the She2p dimer [19] and not on the She2p tetramer [33]. The C-terminal six residues deleted in She2p (ΔC) are shown as a dotted, black line with the label “C.” The protruding helix E is shown in blue and was deleted in She2p (ΔhE). Red and green regions close to helix E depict residues mutated in She2p (F195A L196A) and She2p (Q197A E198A I199A), respectively. (B) shows the same structure rotated by 90° around the vertical axis. (C) shows a close-up of (A), whereas (D) shows the same close-up as (C) rotated by 90° around the horizontal axis. The dotted line in (D) indicates a loop region not resolved in the published crystal structure that was mutated in She2p (F183A D184A G185A) and in She2p (T191A D192A).

In order to obtain further details on this minimal ternary complex, we performed UV cross-linking experiments followed by RNase treatment, trypsin digestion, enrichment via TiO_2_, and mass-spectrometric analysis. This recently developed technique [37],[38] allows for an unambiguous identification of protein fragments that directly contact RNA. By using this technique, we identified one peptide in She2p and one peptide in She3p (334–425) that were cross-linked to *ASH1* E3-51 RNA (Figures 4F and S7). In She2p the cross-linked region is part of a small helix, termed helix E, which protrudes at right angles from the body of the structure (Figure 5A–D) [19],[33]. The cross-linked peptide in She3p contains amino acids that are part of the 20 amino acids long fragment identified in this study to be required for synergistic RNA binding with She2p (residues 334–353; Figure 4D–E). Thus, the cross-linking/mass-spectrometry experiments assign a direct molecular function, i.e. RNA binding, to two distinct regions within the ternary complex.

### Mutational Analysis of She3p

Knowing that the C-terminal 92 amino acids of She3p are functionally important, we mutated conserved residues in this region and tested whether synergistic RNA binding with She2p is affected. Two sets of mutations have already been described in the C-terminus of She3p to affect *ASH1*-mRNA localization in vivo [36]. The first of these mutant proteins, She3p (S343E S361E), did not show a significant defect in synergistic RNA binding (Table 1 and Figure S8A,G), whereas the other mutant She3p (S348E) showed a slight reduction in the synergism (Table 1 and Figure S8B,F). Since the observed defect is rather weak, it seems likely that both sets of mutations also affect a different step during RNA localization.

We also generated two additional point mutations at conserved amino acid positions of She3p. Whereas She3p (R341E) did not show any defect in vitro (Table 1 and Figure S8C), the mutant She3p (L364A V367A) showed significantly reduced synergistic RNA binding with She2p (Table 1 and Figure S8D). Further analysis showed that She3p (L364A V367A) alone exhibits impaired She2p binding but wild-type-like RNA binding (Table 1 and Figure S8H,I). Thus, we identified a region in She3p required for direct She2p interaction and synergistic RNA binding with She2p.

### Deletion of the Helix E or of the Very C-Terminus of She2p Impairs Synergistic RNA Binding with She3p

We also generated mutant versions of She2p and tested them for interaction with She3p and RNA. Two structural regions of She2p show high sequence conservation [19] but have not yet been assigned to a specific function. One region is the helix E of She2p that we already identified to UV-cross-link to zip-code RNA (Figures 4F and 5B–D), indicating that it is part of the RNA-binding surface of She2p. The second conserved region is the protease-sensitive, very C-terminus of She2p (Figure 5A). In the modeled tetrameric structure of She2p, the C-terminus is located at the dimer-dimer interface, close to the previously assigned RNA-binding surface [33]. We created She2p deletion mutants of both regions and confirmed their integrity by size-exclusion chromatography (Figure S9A).

**Figure 6:**
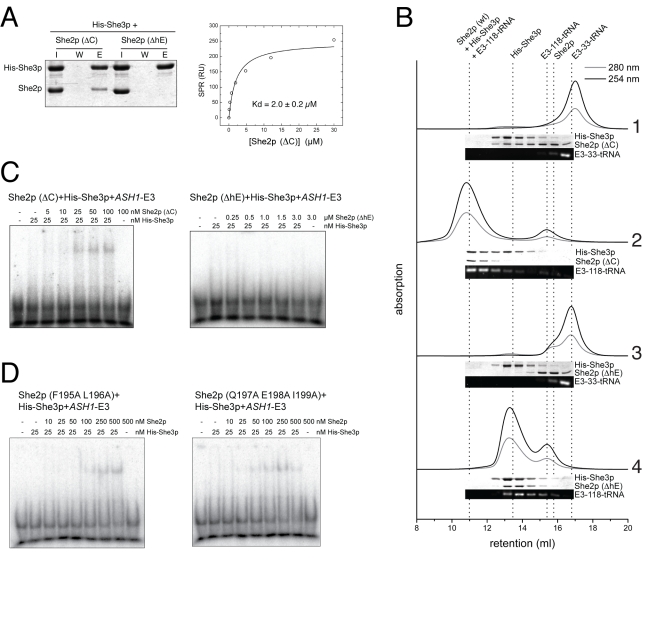
Complex formation and RNA binding by She2p mutants. (A) Binding of She2p mutants to She3p was investigated by pull-down (left) and surface plasmon resonance (right) experiments. In pull-down experiments, She2p (ΔC) but not She2p (ΔhE) co-purified with immobilized His-tagged She3p (I, input; W, final wash; E, elution). In steady-state surface plasmon resonance experiments, She2p (ΔC) bound to surface-coupled She3p with a Kd of 2.0±0.2 µM (mean and deviation of two independent experiments). (B) In analytical size-exclusion chromatography, She2p (ΔC) (chromatogram 2) but not She2p (ΔhE) (chromatogram 4) eluted as high-molecular weight complex with She3p and E3-118-tRNA. Please note that She3p co-migrates with the She2p (ΔhE):E3-118-tRNA co-complex, but no ternary complex is formed (Figure S9C and dotted line indicating the elution volume of the ternary complex). No ternary complexes were observed with E3-33-tRNA (chromatogram 1 and 3). Corresponding fractions were analyzed by SDS-PAGE and agarose gel electrophoresis and are shown below each chromatogram. Dotted lines mark the retention volumes of the indicated components. (C) Also in EMSAs, these She2p mutants show defects in ternary complex formation. Compared to wild-type She2p, an at least 5-fold higher amount of She2p (ΔC) (25 nM) was required for the formation of ternary complexes, whereas She2p (ΔhE) entirely failed to assemble complexes with *ASH1*-E3 RNA and She3p. (D) She2p versions harboring point mutations around the helix E were tested by EMSA. The mutants She2p (F195A L196A) and She2p (Q197A E198A I199A) showed defects in synergistic RNA binding with She3p. A summary of all She2p mutants tested is shown in Table 1.

When performing pull-down experiments with His-tagged She3p, She2p (ΔC) could be co-eluted (Figures 6A, S9B, and Table 1; compare with wild-type She2p in Figure 2A). In contrast, no binding was observed with She2p (ΔhE) (Figures 6A, S9B, and Table 1). SPR with amine-coupled She3p showed almost wild type-like binding by She2p (ΔC) (2.0±0.2 µM), whereas no binding was observed with She2p (ΔhE) even at experimental concentrations of 150 µM (Figure 6A; compare with Figure 2B). We conclude that She2p requires its protruding helix E for the interaction with She3p. In contrast, the very C-terminus of She2p is dispensable for She3p binding.

Next we assessed ternary complex formation with both She2p mutants by size-exclusion chromatography. Whereas She2p (ΔC) still forms stable complexes with She3p and E3-118-tRNA, no ternary complexes assembled with She2p (ΔhE) (Figures 6B and S9C). When using the control RNA E3-33-tRNA, no ternary complex was observed for any of these proteins.

We also used EMSAs to study synergistic RNA binding of the She2p mutants with She3p. Compared to wild-type She2p, the She2p (ΔC) mutant showed an about 5-fold reduced complex formation with She3p and the E3 zip-code element (Figure 6C; compare with Figure 3C). Since the binary interaction of She2p (ΔC) with She3p is largely unaffected (compare Figure 6A with Figure 2B), deletion of the C-terminus mainly impairs the synergistic RNA binding with She3p. No mobility shift was observed with She2p (ΔhE) (Figure 6C), which confirms that helix E plays an essential role in the formation of the ternary complex. We also analyzed ternary complex formation of these She2p mutants with the *EAR1* zip-code RNA and observed defects comparable to the ones obtained with *ASH1*-E3 RNA (Figure S9D, compare with Figure 3D).

Finally, we analyzed the effects of both mutations on the RNA binding of She2p alone. Since in our hands RNA binding by She2p is too transient for detection by EMSAs (Figure S5A,B), these interactions were tested by filter-binding assays [19],[33]. In these experiments, both mutants showed reduced binding to zip-code RNAs (Figure S9E). Interestingly, She2p (ΔhE) still bound unrelated RNAs like wild-type She2p (Figure S9E), suggesting a role of helix E in specific RNA recognition. Defects in RNA-binding by She2p (ΔC) were much more pronounced for *ASH1* zip-code elements than for *EAR1* mRNA. These observations are consistent with respective defects in ternary complex formation. Furthermore, they demonstrate that selective reduction of RNA binding, as observed for She2p (ΔC), impairs synergistic RNA binding with She3p.

### Point Mutations around the Helix E of She2p Impair Synergistic RNA Binding with She3p

Deletion of the helix E of She2p abolishes synergistic complex formation. Because this deletion constitutes a considerable alteration of the overall shape of the protein, we generated more subtle point mutations around this region (Figure 5A–D). Whereas the mutant versions She2p (E183A D184A G185A) and She2p (T191A D192A) affect the flexible loop region at the tip of helix E (dotted line in Figure 5D), She2p (E195A L196A) and She2p (Q197A E198A I199A) altered amino acids that directly interact with helix E and are part of a joint surface landscape with this helix (Figure 5C,D). She2p versions with mutations in the flexible loop region did not show any defect in synergistic RNA binding with She3p (Table 1 and Figure S10A,B). In contrast, the helix E–affecting mutants She2p (E195A L196A) and She2p (Q197A E198A I199A) displayed reduced synergistic RNA binding with She3p (Figure 6D and Table 1; compare with Figure 3C). With both mutants the interaction with She3p was abrogated and RNA binding was moderately reduced (Figure S10C–E). We used circular dischroism spectroscopy to confirm that both mutant proteins adopt the alpha-helical fold observed for the wild-type protein (Figure S10F).

### Deletion of Helix E or the C-Terminus in She2p Abolishes mRNP Assembly In Vivo

**Figure 7:**
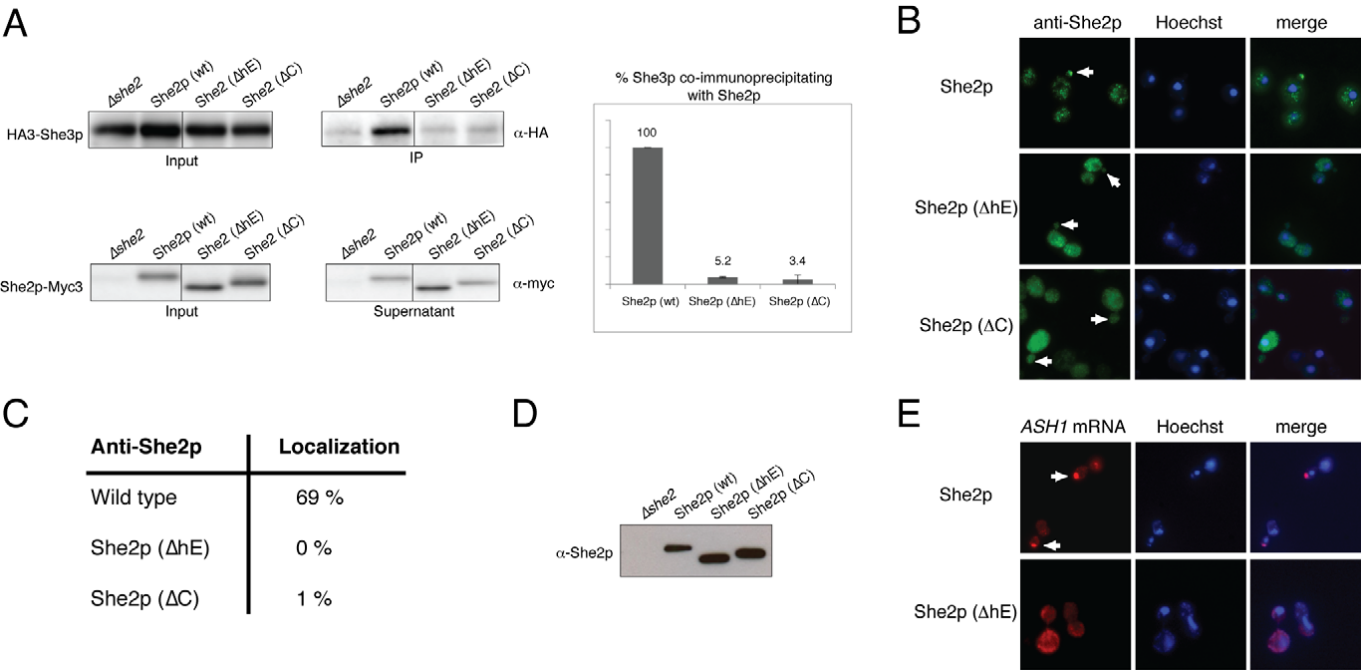
Assessment of *ASH1*-mRNP function in yeast cells. (A) Analysis of mRNP assembly by co-immunoprecipitation of myc-tagged She2p and HA-tagged She3p with anti-myc antibody. Transport-complex assembly was monitored by Western blotting against HA-tagged She3p. The relative amount of co-immunoprecipitated She3p was quantified from two independent blots. Wild-type She2p efficiently co-immunoprecipitated She3p. In contrast, complex assembly was almost abolished in cells expressing either She2p (ΔC) or She2p (ΔhE). Input fractions show equivalent expression levels of HA3-She3p in She2p-mutant strains. Likewise, the She2p mutants were expressed at levels comparable to wild-type She2p. (B) Representative images of immunostaining against wild-type and mutant forms of She2p. Arrows mark the bud tip, to which She2p usually localizes. (C) Immunostaining reveals that She2p localized to the bud tip in 69.2% of wild-type She2p-expressing cells, but not above background levels in cells that either expressed She2p (ΔC) or She2p (ΔhE) (≤1% localization; *n* = 3×>100 budding cells). (D) Western blot analysis with anti-She2p antibody shows that cells expressed the mutant proteins She2p (ΔhE) and She2p (ΔC) at levels comparable to wild-type She2p. (E) *ASH1*-mRNA localization in response to She2p (ΔhE) mutant expression. In wild-type cells, *ASH1* mRNA efficiently localized to the bud tip, whereas in She2p (ΔhE)-expressing cells no localization was observed (*n*>100 cells). Left panels show in situ hybridization against *ASH1* mRNA, middle panels show Hoechst nuclear staining, and right panels display merged images. Arrows indicate *ASH1*-mRNA localization at the bud tip.

To analyze how She2p mutations affect the formation of stable translocation complexes in vivo, we performed co-immunoprecipitation experiments of Myc-tagged She2p followed by Western blot analyses against HA-tagged She3p. Experiments were performed in *she2Δ* background. Wild-type She2p-Myc efficiently co-precipitated She3p (Figure 7A, IP lanes), indicating that assembled translocation complexes can be detected. In contrast, co-immunoprecipitation with She2p-Myc (ΔhE) or She2p-Myc (ΔC) did not show any She3p interaction above background levels (Figure 7A). Thus, these experiments together with our reconstitution studies show that synergistic mRNA binding of She2p and She3p is important for the assembly of mRNPs in vivo.

### Impaired Ternary Complex Formation Also Abolishes She2p-Bud Localization

We also investigated She2p (ΔhE) localization by antibody staining in *she2*Δ cells. As expected, She2p (ΔhE) completely failed to localize to the bud tip (0%, *n*>300 cells; Figure 7B–D). We further tested localization of the C-terminally truncated version of She2p, which moderately affects ternary complex formation in vitro but exhibits wild-type-like binding to She3p (Table 1). The She2p (ΔC) mutant also failed to localize to the bud tip above background levels (i.e. 1.0%, *n*>300 cells; Figure 7B–D).

### Impaired Ternary Complex Formation Abolishes *ASH1*-mRNA Localization In Vivo

In addition, we transformed a *Δshe2* strain with a plasmid expressing She2p (ΔhE) under its endogenous promoter and assessed *ASH1*-mRNA localization by in situ hybridization. In contrast to wild-type She2p, we did not observe any *ASH1*-mRNA localization in response to She2p (ΔhE) expression (Figure 7E; *n*>100 cells). Thus, disruption of ternary complex formation as observed in vitro also results in abolished *ASH1*-mRNA localization in vivo.

### She3p Does Not Shuttle into the Nucleus

**Figure 8:**
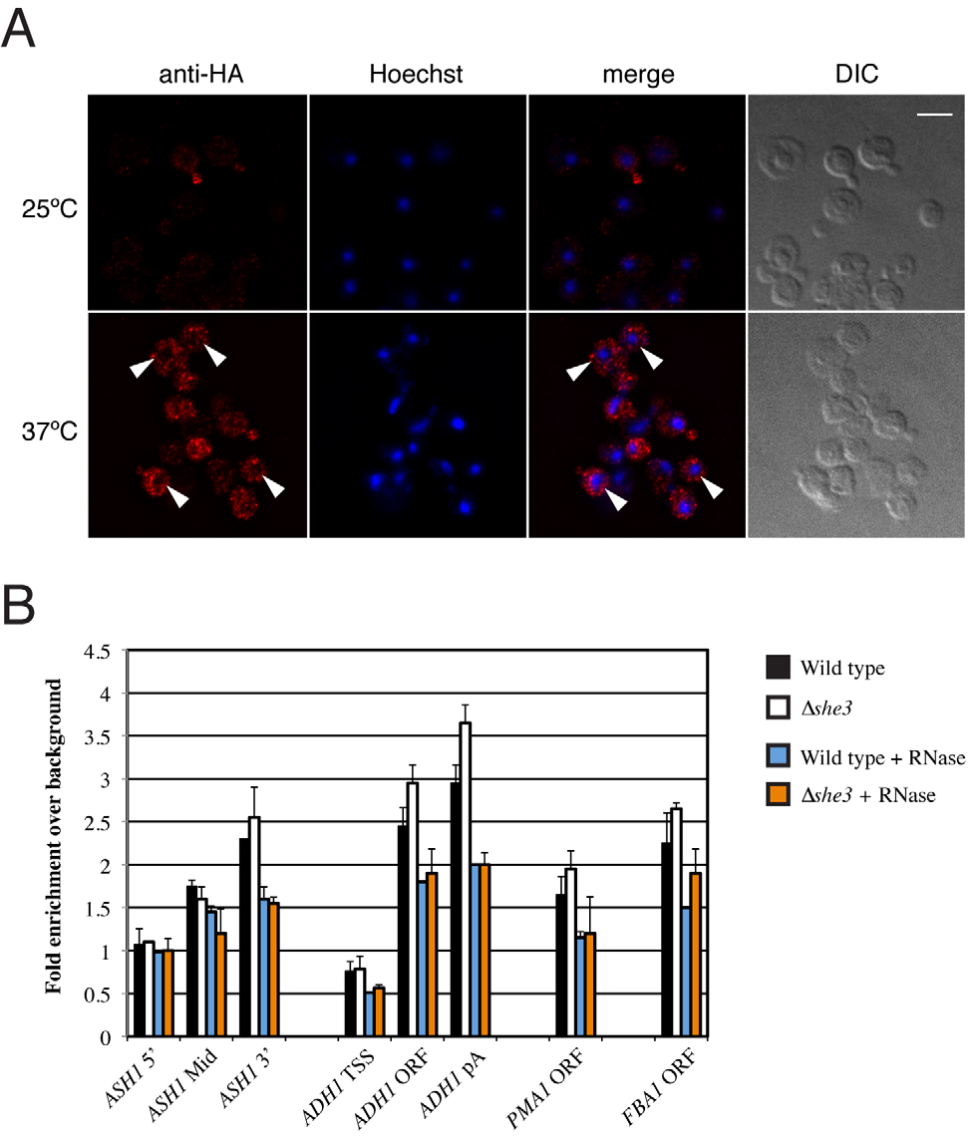
She3p does not play a role in the nucleus. (A) Immunofluorescence stainings of HA-tagged She3p in the temperature-sensitive nuclear export mutant *mex67-5*. At permissive temperature (25°) She3p was localized to the bud tip (upper panels). At restrictive temperature (37°) nuclear export is blocked, but She3p still remained in the cytoplasm (lower panels; position of nuclei marked by arrowheads). It indicates that She3p does not shuttle into the nucleus. At restrictive temperature, She2p is trapped in the nucleus and mRNA as well as Myo4p localization is abolished [16], which is consistent with the diffuse cytoplasmic distribution of She3p. Bar: 5 µm. (B) RNase sensitive She2p occupancy at different genes in wild-type and mutant yeast cells lacking She3p. She2p is recruited co-transcriptionally to genes coding for localized and non-localized transcripts, as it was reported recently [20]. She2p occupancy increases towards the 3′-end of genes. There are no significant differences of She2p occupancy in wild-type and Δ*she3* cells (two-tailed *t* test *p* values>0.05 for all genomic regions tested). An RNase treatment prior to the immunoprecipitation step led to a clear reduction of She2p occupancy, indicating an association with nascent mRNA. The most severe effect was observed at the 3′-end of genes (*ASH1*, *ADH1*: 30%–40% of reduction). Again, no significant differences could be observed between wild-type and mutant yeast cells. RNase treatment affected She2p occupancy at genes coding for localized and non-localized transcripts in a similar way. ChIP occupancies are shown for three different regions of *ASH1* (all primer pairs are located within the ORF region) and *ADH1*, as well as for a single region of *PMA1* and *FBA1* as indicated on the *x*-axis. The fold enrichments over an ORF-free heterochromatic region on chromosome V are shown on the *y*-axis. Error bars express the standard deviation from two independent experiments of biological replicates. TSS, transcription start site; ORF, open reading frame; pA, polyadenylation site.

A current model of *ASH1*-mRNP assembly suggests an early assembly of She2p with *ASH1* in the nucleus [7],[16],[20],[21]. Although previous studies did not hint at a nuclear role of She3p [17],[18],[21]–[23],[36], this has not been tested directly. It is therefore a remaining possibility that the ternary complex also plays a functional role in the nucleus. A well-established way of testing nuclear shuttling of proteins that travel together with mRNA is the use of the temperature-sensitive nuclear export mutant *mex67-5^ts^*. At restrictive temperature, shuttling proteins such as She2p accumulate in the nucleus [16]. Immunofluorescence microscopy with *mex67-5^ts^* cells revealed wild-type-like localization of She3p at permissive temperature (Figure 8A). Also at restrictive temperature She3p remained in the cytoplasm, indicating that it does not shuttle into the nucleus. Because at restrictive temperature She2p and other shuttling factors are retained in the nucleus, mRNA localization is abolished [16]. In addition, transport factors like Myo4p and She3p become delocalized in the cytoplasm (Figure 8A) [16], serving as an internal control for the efficient block of nuclear export. In summary, this experiment confirms the previous assumption that She3p is an exclusively cytoplasmic protein. The finding also supports our notion that the highly specific ternary complex only forms in the cytoplasm during the assembly of the mature transport complex.

### She3p Does Not Contribute to Co-Transcriptional Recruitment of She2p

Recent chromatin-immunoprecipitation (ChIP) experiments showed that She2p associates co-transcriptionally with RNA polymerase II [20] in a gene-unspecific manner. A subsequent comparison of She2p ChIP occupancy in presence or absence of RNase also indicated a selective co-transcriptional binding of She2p to localizing mRNAs. Our studies on She3p localization using *mex67-5^ts^* strains yielded no signs of nuclear accumulation of She3p (Figure 8A). It is possible, however, that a minor fraction of She3p enters the nucleus, which escapes visualization by immunostaining. If She3p is present in the nucleus, the ternary complex described in this study should also form and stabilize co-transcriptional RNA binding of She2p.

In order to test this possibility, we performed ChIP experiments with wild-type and Δ*she3* cells. A comparison of the She2p occupancy at the *ASH1* locus and three control loci in wild-type cells and in Δ*she3* cells showed no significant difference (Figures 8B and S11). As previously shown by Shen and colleagues [20], treatment with RNase resulted in a reduced enrichment at the *ASH1* locus (Figures 8B and S11). However, in contrast to Shen and colleagues, we also observed an RNase-dependent reduction of She2p recruitment to genes, which do not encode for localizing mRNAs (Figures 8B and S11). This RNase-dependent reduction of She2p occupancy is the same in wild-type and Δ*she3* cells. In summary, these data show that She3p does not contribute to co-transcriptional She2p recruitment and is therefore unlikely to play an active role in the nucleus.

## Discussion

To date, all components of the *ASH1* mRNP including the core factors connecting mRNA to the myosin motor have been identified [4],[8],[39]. However, largely due to the lack of quantitative interaction studies, the molecular mechanisms leading to specific mRNA recognition and mRNP assembly remain ambiguous. To our knowledge, we performed the first in vitro reconstitution of the core of an mRNA-transport complex. We followed the path of mRNP assembly from the nucleus to the cytoplasm and assessed binding affinities, specificities, and synergisms in complex assembly. Key findings were confirmed by complementing in vitro and in vivo experiments.

Since She2p binds *ASH1* mRNA co-transcriptionally and escorts it until it is anchored at the bud tip [16]–[18],[21], it had been assumed that this protein is responsible for the specific incorporation of zip code containing mRNAs into localizing mRNPs. We found that neither She2p nor Puf6p, which is the other *ASH1*-E3 element-interacting protein present in the nucleus, bind zip-code RNAs with high specificity (Figure 1A–C). Because in a previous study a more specific recognition of *ASH1*-E3 RNA by Puf6p had been suggested [29], we performed additional control experiments to ensure that Puf6p is folded and active in our experiments (Figure S1). Whereas RNA-binding assays with unlabeled competitors are virtually identical in both studies, we also observed strong Puf6p binding to unrelated control RNAs in standard EMSAs. Although we arrive at a somewhat different estimation of the binding specificity of Puf6p, it seems clear from both studies that RNA binding by Puf6p is less specific than by other Pumilio/FBP family members [40]. It might be that Puf6p requires an additional co-factor to achieve the specificity reported for its in vivo function.

Previous immunoprecipitation experiments with yeast extracts showed that the Puf6p:She2p complex is RNase-resistant, suggesting that both proteins might interact directly [21]. With recombinant proteins, we recapitulated an RNA-mediated interaction of Puf6p and She2p, but not a direct protein-protein binding (Figure 1E). These results suggest that *ASH1* mRNPs are rather RNase-insensitive, stable particles that allow for co-purification of both factors even after RNase treatment.

**Figure 9:**
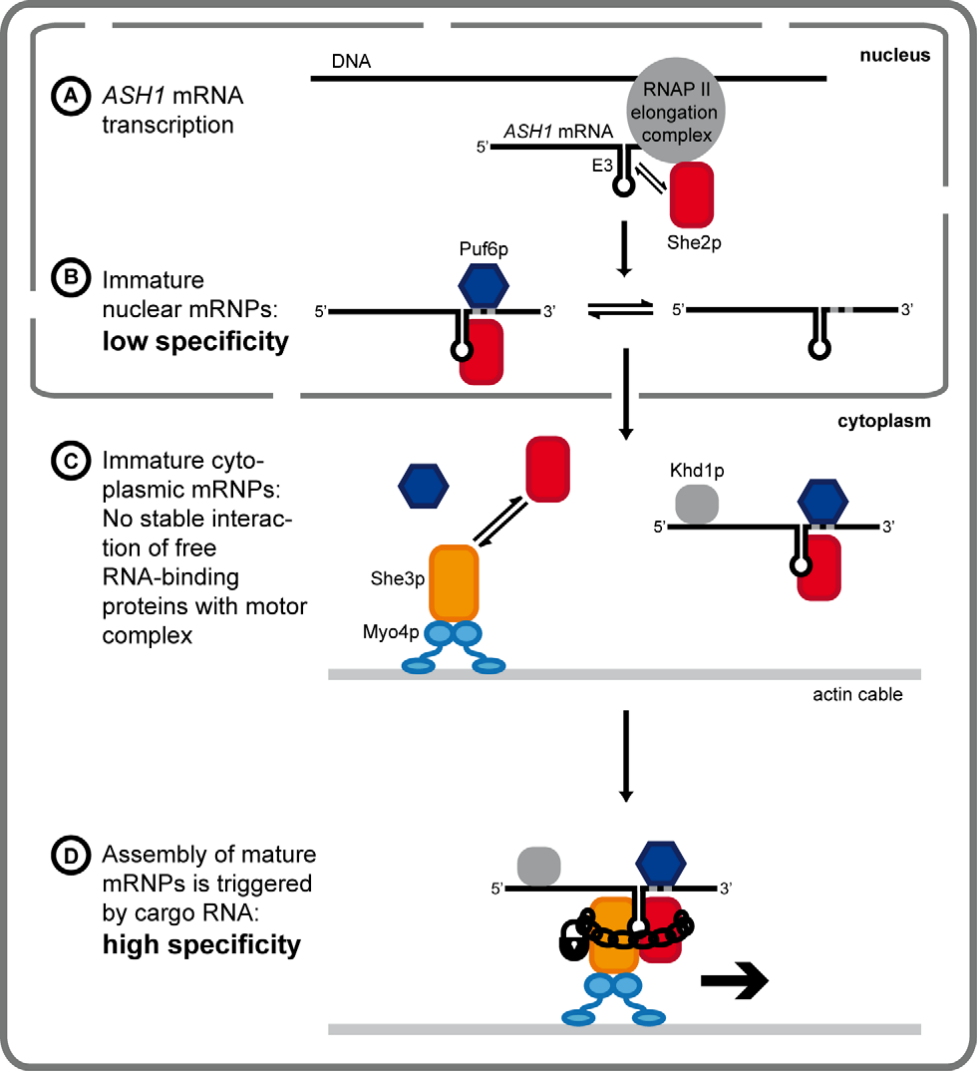
Model for the sequential assembly of *ASH1*-transport complexes in yeast. Solid line shows cell boundaries and dotted line indicates the nuclear envelope. (A) She2p is co-transcriptionally loaded on nascent mRNA. (B) She2p and Puf6p bind in the nucleus with modest specificity to *ASH1* mRNA. (C–D) Following nuclear export, She2p and *ASH1* mRNA are bound by Myo4p-associated She3p to form a stable and specific complex. Puf6p and Khd1p are also associated with the cytoplasmic complex and mediate translational repression during transport. After transport, the complex is anchored at the bud tip and translation is activated [4] (not depicted). The model combines our results with previous findings.

When performing in vitro RNA-binding assays with Puf6p and She2p, we also observed that their combination failed to bind synergistically or with higher specificity to zip-code RNAs (Figure 1D). Together, these experiments suggest that a complex consisting of *ASH1* mRNA, She2p, and Puf6p is able to assemble in the nucleus with moderate specificity (Figure 9). Although unlikely, we cannot exclude the requirement of other factors not yet implicated in the assembly of specific nuclear mRNPs.

In the cytoplasm, She3p is thought to link the RNA:She2p complex to the myosin motor Myo4p [17],[18]. The prevailing model proposes that besides Myo4p, the translationally repressed *ASH1*-E3 complex harbors She2p, She3p, and Puf6p. To date, it had not been shown that such a complex really assembles on the RNA. In our in vitro assays, we reconstituted this Puf6p-containing complex in an *ASH1*-E3 RNA-dependent manner (Figure S4). Whether Khd1p behaves in a similar manner remains to be shown.

A previous study reported that *E. coli*–expressed, crude-purified GST-She3p causes a supershift in an EMSA with GST-She2p and *ASH1*-E3 RNA [17]. From this single experiment, it remained unclear whether this supershift is based on protein-protein or protein-RNA interactions and whether the presence of She3p has implications on RNA-binding affinity and specificity. Diverging models have been proposed on the function of She3p [17],[18],[36], including a scenario where She2p is not required for cytoplasmic *ASH1*-mRNA transport [36].

When assessing molecular interactions of She3p, we first identified a direct and specific binding to She2p with a Kd of 1.6 µM (Figure 2A,B). This interaction suffices to explain the previously described supershift by She3p in EMSAs [17]. It is also consistent with a previous report where She3p was immunoprecipitated with a She2p mutant that lacks RNA-binding capacity [41]. However, the modest affinity and transient nature of the interaction between She2p and She3p and the low RNA-specificity of She2p appears insufficient to explain the specific localization of *ASH1* mRNA observed in vivo.

More surprisingly, we identified the myosin adapter She3p as a new, rather unspecific RNA-binding protein (Figure 3A,B) with affinities comparable to She2p and Puf6p. Database searches failed to identify any known RNA-binding motif in She3p. However, the most important finding for a mechanistic understanding of specific cargo recognition and mRNP assembly was our observation that She2p and She3p together form a highly specific ternary complex with all zip-code elements of localizing mRNAs tested in this study. EMSAs demonstrated that the She2p:She3p complex has at least 60-fold higher Kds for zip-code RNAs over unrelated RNA stem-loops (Figures 3C–E and S5F–H). In addition, this RNA interaction stabilizes the rather weak binary She2p:She3p complex (Figure 2B) to a similar extent. Since for the control HIV-1 TAR RNA no binding was observed even at the highest experimental concentration (Figure 3E), the true difference in Kd might be even higher. These findings were confirmed by a total of 12 mutations in She2p and She3p, in which for seven of them an impaired complex formation was observed (Table 1). Two of these She2p mutants were also tested in vivo, where they showed a total loss of RNA localization (Figure 7). Interestingly, four of the mutations in She2p and She3p with a defect in synergistic complex formation also showed defects in both binary interactions, RNA and protein binding. This observation suggests that both interactions are spatially and mechanistically intertwined to allow for the synergism described in this study. This conclusion is further supported by UV cross-linking experiments, which demonstrate that She2p and She3p both directly bind to RNA in the ternary complex (Figures 3F, 4F, and S7).

No known RNA-binding motif can be identified in She3p. We nevertheless found a C-terminal fragment of 92 amino acids to be sufficient for synergistic RNA binding with She2p. Within this 92 amino acids long fragment, we mapped residues that are of functional importance for the synergism. Our subsequent UV cross-linking/mass-spectrometry experiment with She2p, RNA, and the C-terminal fragment of She3p confirmed that a functionally important subfragment of 20 residues is indeed involved in RNA binding (Figures 4F and S7). These cross-linking experiments also showed that the helix E of She2p directly interacts with RNA. The latter finding confirms our observations on the functional importance of the helix E and assigns a direct molecular function, i.e. RNA binding, to this protein region. A surface plot of all She2p-surface regions that are important for RNA binding shows a large continuous RNA-interaction surface (Figure S12).

In a recent study, a She3p-dependent *ASH1* mRNA-transport system was reported in *Candida albicans* [42]. Although in this yeast species no clear She2p homolog could be identified, *ASH1* mRNA is transported in a fashion similar to *S. cerevisiae*. Thus, the question arose of how cargo binding is achieved. Our identification of *S. cerevisiae* She3p as an RNA-binding protein suggests that *C. albicans* She3p could mediate at least part of the RNA binding for mRNP assembly and transport. A sequence alignment of She3p from different yeast species reveals that the C-terminal half of this protein shows great differences between species with and without She2p in their genomes (Figure S13). As shown in this study, the C-terminal part of *S. cerevisiae* She3p binds to She2p (Figure 4B) [17],[18] as well as to RNA (Figure 4C) and is required for synergistic RNA binding with She2p (Figure 4D–F). It therefore appears likely that the She2p-lacking species have optimized the C-terminal She3p sequence for an interaction with a different RNA-binding protein or even for a more specific RNA-binding by She3p itself.

Previous studies suggested that She3p acts only in the cytoplasm [17],[18],[21]–[23],[36]. However, this assumption had not been rigorously tested before. We used a nuclear export mutant to show that She3p indeed does not shuttle into the nucleus (Figure 8A). To scrutinize whether a small sub-fraction of She3p might play a role in the nucleus, we also analyzed the recently reported co-transcriptional recruitment of She2p to chromatin by ChIP experiments [20]. Since we observed no significant difference of She2p occupancy in wild-type and Δ*she3* strains, a nuclear role of She3p could be further excluded (Figure 8B).

Shen et al. also reported a reduction of She2p chromatin binding in ChIP experiments after RNase treatment selectively at open reading frames (ORFs) of localizing mRNAs. The authors concluded that part of the chromatin-associated She2p interacts selectively with localizing mRNAs already during transcription. However, we could not confirm this observation. Although we also detected a reduction of She2p-dependent enrichment after RNase treatment, this effect was observed for all transcripts (Figure 8B). Thus, our ChIP experiments suggest rather unspecific She2p association with nascent transcripts. They further indicate that She3p does not play a functional role in the nucleus.

In summary, our and previous data suggest the following model: First, She2p binds co-transcriptionally to RNA polymerase II and to nascent transcripts (Figure 9A). After transcription, nuclear mRNAs are bound by She2p and Puf6p with only limited specificity (Figure 9B), followed by a nuclear export of both proteins together with mRNAs (Figure 9B,C). In the cytoplasm, She2p and localizing mRNAs form a highly specific co-complex with myosin-bound She3p (Figure 9C,D). This transport complex mediates the translocation of cargo mRNAs to the bud cell (Figure 9D), where after anchoring at the bud tip translation is activated.

On one hand, She2p and She3p function together exclusively in the cytoplasm to select zip-code RNAs. On the other hand, the mRNA cargo itself substantially stabilizes the She2p:She3p interaction. Because this interaction brings together the She2p-dependent pre-mRNP with the cytoplasmic motor complex, we conclude that it is the mRNA cargo itself that triggers joining of all components into the mature transport complex. This interpretation is fully consistent with the observation that RNase-treated *ASH1*-mRNPs do not have motile activity in vitro [27]. We propose that coupling of specific mRNA recognition and assembly of stable transport complexes constitutes a critical quality control step to ensure that only target mRNAs are transported.

Previous publications reported only moderate in vitro selectivity for localizing mRNAs in yeast and *Drosophila* (e.g. between 3- and 7-fold higher Kd for localizing RNAs) [6],[7]. Thus it remained ambiguous what difference in affinity to localizing and non-localizing RNAs might be required for specific mRNA transport and how highly specific mRNP assembly is achieved in vivo. In comparison, the ternary complex formation described in this study shows an unprecedented selectivity for zip code containing RNAs. In our understanding, this observation gives a more realistic example for the cargo specificity required for mRNA localization. It also demonstrates that co-complexes of transport factors might play a much more important role in the recognition of transcripts than previously assumed. Last but not least, our finding that the cargo RNA itself triggers the incorporation of all protein-core factors into one mature transport complex provides a new mechanistic paradigm for the assembly of RNA-localization complexes.

An important question arising from this study is whether synergistic binding to RNA cargo is a more general feature in eukaryotes. For instance, during the oocyte-to-embryo transition of *Drosophila* development, the RNA-binding protein Staufen is involved in the localization of *bicoid* mRNA. In vitro, Staufen yielded strong binding to specific as well as to control RNAs with extensive secondary structures [43]. In contrast, in vivo injection experiments in *Drosophila* embryos showed that Staufen-containing mRNPs only form and localize efficiently when its native target, the *bicoid* 3′UTR, is injected [44]. Thus, it might well be that those *Drosophila* complexes also require mRNP assembly for specific mRNA binding and localization.

In the past, in vitro reconstitution of molecular assemblies has been very successful to provide new insights into biological processes as diverse as transcription and membrane fusion [45]. By showing that specific mRNA recognition and assembly of stable cytoplasmic transport complexes is coupled, we demonstrate that in vitro reconstitution is also well suited to advance our mechanistic understanding of mRNA-transport.

## Materials and Methods

### Plasmids and Yeast Strains

Detailed information on plasmids, oligonucleotides, and yeast strains are found in Tables S1–S3. Information on cloning and generation of yeast strains are summarized in Text S1.

### Protein Expression and Purification

She2p and Puf6p were expressed as GST-fusion in *E. coli* BL-21 Star (DE3) cells (Invitrogen) and purified using standard techniques [19], including affinity, ion-exchange, and size-exclusion chromatography columns. His-She3p (334–425) and His-She3p (354–425) were expressed in fusion with either GST or MBP in *E. coli* BL-21 Star (DE3) cells and purified via affinity and size-exclusion chromatography. The GST-tag was cleaved with PreScission protease (GE Healthcare) during purification, unless stated otherwise. Full-length His-She3p and His-She3p point mutants were co-expressed with She2p using the Bac-to-Bac system (Invitrogen). Recombinant baculovirus was amplified in Sf21 cells and used to infect High Five insect cells. Cells were cultured for 60–70 h. After sonication, His-She3p was purified using HisTrap, HiTrap Q, HiTrap Heparin, and Superose 6 10/300 GL columns (GE Healthcare). She2p and nucleic acids were removed by extensive washing with 1 M NaCl-containing buffer. At the last step of purification, RNA-binding proteins were assessed by measuring the ratio of A_260_ to A_280_ to exclude contaminations by nucleic acids. Myo4p tail was purified as previously described [46].

### RNA Preparation

RNAs for filter-binding assays and EMSAs were produced by either in vitro transcription or total chemical synthesis (Table S4). In vitro–transcribed RNAs were purified using native PAGE. For pull-down assays and analytical size-exclusion chromatography experiments, *ASH1*-E3 RNA was fused to the *E. coli* initiator tRNA(Met) (E3-33-tRNA, E3-77-tRNA, and E3-118-tRNA; Table S4), expressed in *E. coli* JM101 cells (New England Biolabs), and purified by ion exchange chromatography essentially as described [34],[35].

### RNA Filter-Binding Assay

RNA filter-binding assays were essentially performed as described [33]. Serial She2p dilutions were incubated with 0.5 nM of radiolabeled RNA in Dot-Blot buffer (pH 7.4) (20 mM HEPES (pH 7.5), 150 mM NaCl, 2 mM MgCl_2_, 2 mM DTT, and 30 µg/ml yeast tRNA). Four-fifths of the reaction mixture was applied on the nitrocellulose membrane and radioactivity retained was measured by phosphoimaging. Kd calculation was performed by plotting the fraction of bound RNA versus the protein concentration and applying the Langmuir isotherm. The following serial dilutions were used: wild type–She2p binding to HIV-I TAR (16 bases, 57 bases) or U1snRNA hairpin II: 0 to 12 µM; She2p-mutant binding to bud-localizing RNAs: 0 to 16 µM; She2p (ΔC)-binding to *ASH1*-E2A element: 0 to 32 µM; She2p-mutants binding to HIV-I TAR (16 bases), U1snRNA hairpin II, poly(A)_20_ RNA: 0 to 32 µM; and She2p-F195A-L196A and She2p-Q197A-E198A-I199A to *ASH1*-E3: 0 to 2 µM. Standard deviations were calculated from at least three independent experiments. To show synergistic RNA-binding effects, serial She2p dilutions (0 to 320 nM for *ASH1*-E3, 0 to 2.67 µM for *EAR1*) were incubated with 25 nM She3p and 0.5 nM ^32^P-RNA in Dot-blot buffer (pH 7.9) and treated as described above. For data analysis, the RNA-binding signal of 25 nM She3p alone was subtracted as background. Signal intensities of She2p:She3p:RNA complexes were plotted against the respective She2p concentrations and normalized to the relative signal intensities of the corresponding She2p:RNA complexes.

### Electrophoretic Mobility Shift Assay (EMSA)

In a 20 µl reaction, protein was incubated with 5 nM of ^32^P-labeled RNA oligonucleotide in HNMD-buffer (20 mM HEPES (pH 7.8), 200 mM NaCl, 2 mM MgCl_2_, 2 mM DTT) supplemented with 4% (v/v) glycerol and 30 µg/ml (EMSAs with She2p and She3p) or 100 µg/ml yeast tRNA (EMSAs with She3p or Puf6p only) for 30 min at 20°C. For competition experiments, excess of unlabeled competitor RNA was added to the reaction after 30 min preincubation and incubated for another 15 min at 20°C. Additionally, HNMD-buffer supplemented with 100 µg/ml yeast tRNA was used. Protein:RNA complexes were resolved by PAGE (native 4% gel in 0.5× TBE running buffer, 90 V for 45 min at 20°C). Gels were scanned with a Storm Scanner and analyzed using the software ImageQuant.

### UV Cross-Linking Experiments

In 20 µl reactions, recombinant She2p and She3p at indicated amounts were incubated with 5 nM ^32^P-labeled *ASH1*-E3-51 RNA for 20 min at 20°C in Dot-Blot buffer (pH 7.9). Subsequently, UV cross-linking was performed for 15 min on ice, using a Spectrolinker XL-1500 (Spectroline) with an average intensity of 2,500 µW/cm^2^. 18 µl of each cross-linked sample were resolved by SDS-PAGE. Gels were scanned and analyzed using the software ImageQuant.

### UV Cross-Linking Experiments Followed by Mass Spectrometric Analysis

100 µg of purified ternary complex (*ASH1*-E3-51 RNA, full-length She2p, and His-She3p (334–425)) in 200 µL HNMD-buffer were cross-linked for 10 min at 254 nm. After digestion of the sample with RNase A, RNase T1, and trypsin, cross-linked peptides were enriched on a TiO_2_ column [37],[38] and analyzed by Nano-LC-ESI-MS. Data analysis for the identification of cross-linked peptides was carried out essentially as described earlier [37]. A detailed description is provided in Text S1.

### Analytical Size-Exclusion Chromatography

Chromatography was performed with a Superose 6 10/300 GL column. 20 µM wild-type or mutant She2p, 30 µM She3p and 8.5 µM RNA (E3-33-tRNA or E3-118-tRNA) were preincubated for 5 min at 20°C. Then 200 µl samples were loaded on the column in HNMD-buffer (flow rate: 0.5 ml/min). Fractions were analyzed by SDS PAGE (Coomassie blue staining) and 2% agarose gels (in 1× TBE) stained with GelRed DNA Stain (Biotium).

### In Vitro Pull-Down Experiments

In a volume of 100 µl, 7.5–10 µM of each protein and, if applicable, 5 µM *ASH1*-E3-tRNA-fusion were incubated in HNMD-buffer with 50 µl resin (nickel sepharose for His-She3p; glutathione sepharose for GST-Puf6p) for 30–60 min at 4°C on a rotating wheel. Binding reactions were pelleted and washed four times with 200–500 µl of HNMD-buffer, followed by a final washing step with 50 µl. For His-tagged She3p, HNMD-buffer for binding and washing was supplemented with 30–50 mM imidazole. Bound proteins were eluted with 50 µl of elution buffer (nickel pull-downs: HNMD-buffer supplemented with 750 mM imidazole; GST pull-downs: 30 mM reduced glutathione). One-tenth of the input, 1/5 of the final wash, and 1/5 of the elution fraction were analyzed by SDS-PAGE and Coomassie blue staining. RNA content was analyzed with 2% agarose gels (in 1× TBE) stained with GelRed DNA Stain (Biotium).

### Surface Plasmon Resonance

All experiments were performed using a Biacore 3000 system with a CM-5 chip (Biacore) in HNMD-buffer. She3p was covalently bound to the chip surface by standard amine coupling. For measurements with She2p (wt) and She2p (ΔC) concentrations from 0.123 to 30 µM were used. She2p (ΔhE) binding was probed with up to 150 µM protein. All binding signals were below 300 response units. Experiments were performed as duplicates and double referencing was applied. Kds were derived from steady-state measurements, applying the Langmuir isotherm.

### Fluorescence Microscopy

In situ hybridization was performed using TexasRed-conjugated antisense oligonucleotides against *ASH1* mRNA [47]. Immunostaining was performed either using polyclonal anti-She2p antibody [7] and goat anti-rabbit antibody coupled to AlexaFluor488 or using 3F10 anti-HA antibody (Roche) directed against She3-6×HA and goat anti-rat coupled to Alexa594. DNA was stained with Hoechst Stain Solution (Sigma). Cells were inspected with an Olympus BX60 or Zeiss CellObserver fluorescence microscope and images acquired on a Hamamatsu OrcaER or Zeiss MRM CCD camera using the Openlab 4.0 software (Improvision) or Axiovision (Zeiss) software. In each experiment, at least 100 budding yeast cells were counted (see text). Expression levels of She2p were analyzed by Western blotting using the monoclonal anti-She2p antibody She2p-1C3 [33].

### Co-immunoprecipitation

Co-immunoprecipitation of Myc-tagged She2p was performed using monoclonal anti-Myc antibody (9E11, Acris Antibodies) coupled to magnetic Protein G beads (Invitrogen) [33]. For quantification, Western blots of two independent experiments were analyzed using the LAS-3000 mini system and Multi Gauge software (FUJIFILM). Background signals from *Δshe2* lanes were subtracted from respective Western blot signals. Subsequently, She2p signals from the supernatant fractions were subtracted from input fractions and used to normalize co-immunoprecipitated She3p against the bead-bound fraction of She2p-Myc3.

### Chromatin Immunoprecipitation (ChIP)

ChIP experiments were performed as previously described [48] with the following exceptions. Yeast strains containing a TAP-tagged version of She2p were grown in 100 ml YPD medium to mid-log phase (OD_600_∼0.8). To increase the fraction of cells with *ASH1* transcription, yeast cultures were treated with nocodazole (final concentration: 15 µg/ml) for 2 h and washed once with 100 ml YPD as described [20] before cross-linking with formaldehyde (final concentration: 1%). The additional RNase treatment step in the ChIP protocol required a reduction of the cross-linking time from 20 to 5 min. For better comparison, ChIP experiments without RNase treatment were treated identically. The RNase treatment was performed essentially as described [49]. Briefly, the chromatin was treated with 7.5 U of RNase A and 300 U of RNase T1 (RNase Cocktail Enzyme Mix; Ambion). After incubating at room temperature for 30 min, immunoprecipitations were performed. Expression levels of She2p-TAP were analyzed by Western blotting using anti-TAP antibody (Sigma) and monoclonal antibody anti-She2p-1C3 [33].

### Quantitative Real-Time PCR (qPCR)

The experiments as well as the analyses were performed as previously described [48],[50]. Input and immunoprecipitated (IP) samples were assayed by qPCR to assess the extent of protein occupancy at different genomic regions. Primer pairs were designed for three different open reading frame (ORF) regions of *ASH1*, a distinct region within the ORF regions of *PMA1* and *FBA1*, the promoter, coding and terminator regions of *ADH1*, as well as for a heterochromatic control region of chromosome V. The PCR efficiencies were determined prior to ChIP experiments and were in the range of 95%–100%. The sequences and the location of the primers used in this study are shown in Table S5.

## Supporting Information

Figure S1 Control experiments to confirm that Puf6p is an intact protein. (A) In size-exclusion chromatography full-length Puf6p eluted as a single, defined peak. No major elution was observed at the void volume of about 8 ml, confirming that Puf6p does not aggregate. The sharp elution peak of Puf6p further indicates a defined conformation of this protein. (B) Circular dichroism spectroscopy with Puf6p reveals a profile typical for alpha-helical proteins. Since Puf6p contains seven Pumilio-homology domains [29], which are highly alpha-helical [51], this profile is expected for correctly folded Puf6p.
(0.09 MB PDF)

Figure S2 Structure predictions of the *ASH1* mRNA variants E3-118, E3-51, and E3-33. Predictions were generated with MC-Fold [52]. Bold lines with dot represent Watson-Crick base pairs, whereas lines indicate non-Watson-Crick base pairs. Grey boxes in E3-118 show the location of the two *PUF*-consensus sequences UUGU [29]. The numbering starts at base one of the *ASH1* mRNA start codon. The zip-code stem loops E3-118 and E3-33 were fused to the anticodon stem of tRNA(Met) [35],[53]. Schemes of tRNAs are not drawn to scale. The complete sequences of the constructs are given in Table S4.
(0.16 MB PDF)

Figure S3 Control experiments for complex assembly with She2p. (A) In pull-down experiments, wild-type She2p did not interact with nickel-sepharose beads. (B) In size-exclusion chromatography experiments, wild-type She2p and She3p eluted as a co-complex in absence of RNA. (C) Size-exclusion chromatography of the reconstituted *ASH1*-E3 mRNA core complex, consisting of *ASH1*-E3 RNA, She2p, She3p, and the interacting Myo4p-tail fragment. Corresponding fractions were analyzed by SDS-PAGE and agarose gel electrophoresis and are shown below the chromatogram.
(0.15 MB PDF)

Figure S4 Reconstitution of a major part of the cytoplasmic *ASH1*-E3 mRNP. (A) In pull-down experiments with immobilized His-She3p, Puf6p and She2p co-eluted in an *ASH1*-E3 zip-code-dependent manner (lanes 1–3; pulled-down proteins are indicated by asterisks). No co-elution of Puf6p was observed when the control RNA *ASH1*-E3-33 was used (lanes 4–6) or in absence of RNA (lanes 7–9). As observed previously, She3p bound to She2p also in absence of RNA. The small amount of *ASH1*-E3-33 RNA eluting with She3p from the nickel sepharose beads (lane 6) most likely resulted from unspecific RNA binding by She3p (see also Figure 3A,B). (B) Pull-down experiments with immobilized GST-tagged Puf6p revealed a complex of Puf6p, She3p, and She2p only in presence of the functional *ASH1*-E3 zip code (lanes 1–3; pulled-down proteins are marked by an asterisk), but not in absence of RNA (lanes 4–6). Please note that She3p always migrates as a double band in SDS gels. In a control experiment with immobilized GST protein, no unspecific binding of She2p, She3p, or RNA was observed (lanes 7–9). (C) Control pull-down experiments with immobilized His-tagged She3p confirmed the formation of a ternary complex consisting of She3p, She2p, and *ASH1*-E3 RNA (lanes 1–3) but did not show an interaction of She3p with Puf6p in the presence of *ASH1*-E3 RNA (lanes 4–6). No unspecific binding of She2p and *ASH1*-E3 RNA to the beads was observed (lanes 7–9). In contrast, Puf6p retained a weak affinity to nickel sepharose, which could not be eliminated by using 50 mM Imidazole in binding and washing buffers. Please also note that the band migrating above His-She3p represents a degradation product of Puf6p (compare lane 4 with 7). I, input; W, final wash; E, elution.
(0.42 MB PDF)

Figure S5 Supporting experiments for studies on synergistic RNA binding by She2p and She3p. (A–B) The She2p:RNA interaction is too transient to be resolved in EMSA experiments. Even at micromolar She2p concentrations, no stable complex with either *ASH1*-E3 (A) or *EAR1* zip-code RNA (B) was detected. (C) The minimal She3p concentration required for detection of *ASH1*-E3 zip code binding in EMSAs was determined to be in the range of 25 nM to 50 nM (position marked by asterisk). (D) This EMSA shows synergistic binding of She2p and She3p on *ASH1*-E3 RNA in direct comparison to the unaffected Puf6p binding in presence of She2p. A shift to larger molecular weight and thus the formation of a ternary complex was only observed for She2p and She3p. Puf6p alone showed strong but unspecific binding (see Figure 1). (E) Western blot against She2p after EMSA confirms the presence of She2p in the ternary complex with She3p and *ASH1-*E3 RNA. (F–I) EMSAs with a constant She3p concentration (25 nM) and varying amounts of She2p show specific ternary complex formation with the *ASH1*-E1 zip code (F), *ASH1*-E2A zip code (G), *ASH1*-E2B zip code (H), and a shortened *ASH1*-E3-51 zip-code RNA (I). The Kds for these complexes were all estimated to be in a similar range and are comparable to specific complex formation with *ASH1*-E3 and *EAR1* zip codes (see Figure 3C,D). Please note that 25 nM She3p seem to be sufficient for weak binding to *ASH1*-E1 (F) and *ASH1*-E2A (G), resulting in the formation of a subtle band shift. However, RNA:She2p:She3p complexes migrated slightly slower in EMSAs and could thus be distinguished from RNA:She3p complexes.
(1.11 MB PDF)

Figure S6 Control experiments for the observed synergistic complex formation of She2p and She3p on zip code containing RNA. (A) Filter-binding experiments confirm the synergistic binding of She2p and She3p to the *ASH1*-E3 (left) and the *EAR1* (right) zip codes. Shown are the raw data obtained by dot-blot experiments with radiolabeled zip-code RNA, various She2p concentrations, and 25 nM She3p whenever indicated. The presence of She3p in the reaction significantly enhanced RNA binding, as determined by an increase in signal intensity at lower She2p concentrations. (B) In the corresponding graphs, relative signal intensities are plotted against the She2p concentrations to visualize the larger amount of zip-code RNA bound by the She2p:She3p complex. Because ternary complex formation does not appear to follow simple reaction kinetics, we refrained from calculating Kd values.
(0.19 MB PDF)

Figure S7 UV cross-linking and subsequent mass spectrometric analysis of RNA-bound protein fragments. The peptide sequence and cross-linked nucleotides as well as identified peptide CID fragments are indicated above each MSMS spectrum. (A) MSMS spectrum of the N-terminal tryptic peptide GPLGSMGNSSNNK (G334–K340) of His-She3 (334–425), including the preceding linker region GPLGSM (underlined), cross-linked to an AAU oligonucleotide without any terminal phosphate. [A] represents the adenosine nucleotide, while [A′] stands for the adenine base. All further RNA-related signals are labeled accordingly. The signal at m/z 768.14 indicates that S10 could be cross-linked to U, as it can be calculated as the y4 peptide fragment with an additional U nucleotide and a loss of H_2_O. The experimental precursor mass 2,163.68 Da equals the sum of calculated peptide and oligonucleotide masses, 1,261.57 Da and 902.17 Da, respectively, within the mass accuracy of the employed Q-ToF instrument. (B) MSMS spectrum of the N-terminal tryptic peptide GPLGSMGNSSNNK (G334–K340) of His-She3 (334–425) cross-linked to a uridine nucleotide. The peptide's N-terminus is carbamylated due to hydrolyzation in urea at elevated temperatures. The sum of the calculated peptide mass (1,304.58 Da) and the mass of a uridine nucleotide (324.04 Da) equals the experimental precursor mass of 1,628.73 Da. (C) MSMS spectrum of the She2 peptide IGSNLLDLEVVQFAIK (I164–K179) cross-linked to a uridine nucleotide. Peptide fragments with neutral loss of ammonia are marked with an asterisk. The experimental precursor mass of 2,081.92 Da equals the sum of calculated peptide and nucleotide mass (1,757.99 Da and 324.04 Da, respectively).
(0.17 MB PDF)

Figure S8 Analysis of She3p point mutants. (A–D) Synergistic binding of His-She3p mutants and wild-type She2p to *ASH1*-E3 RNA was analyzed by EMSAs. Synergistic RNA binding was not significantly reduced for She3p (S343E S361E) (A) and She3p (R341E) (C) as compared to the wild-type (Figure 3C). She3p (R348E) (B) showed a slightly reduced and She3p (L364A V367A) (D) a strongly reduced synergistic RNA binding. (E–H) Binary interactions of His-She3p mutants with *ASH1*-E3 RNA were analyzed by EMSAs. RNA binding of She3p (R348E) (F), She3p (S343E S361E) (G), and She3p (L364A V367A) (H) was comparable to wild-type She3p (E). (I) Interactions between His-tagged She3p mutants and wild-type She2p were analyzed by in vitro pull-down with nickel sepharose. She2p binding to She3p (S348E) and She3p (S343E S361E) was comparable to wild-type She3p. In contrast, She3p (L364A V367A) did not interact with She2p.
(0.79 MB PDF)

Figure S9 The effect of She2p mutations on RNA binding and complex formation. (A) Elution profiles from preparative size-exclusion chromatography (Superose 12 column) show that wild-type She2p and She2p (ΔhE) eluted at the same volume, whereas She2p (ΔC) showed a retarded elution. Thus, deletion of the C-terminus appears to slightly affect She2p tetramerization without completely disrupting it (for further details, see [33]). The sharp peaks of all She2p fragments confirm protein integrity. (B) Control pull-down experiments show that neither She2p (ΔC) nor She2p (ΔhE) bound to nickel-sepharose beads. (C) She2p (ΔhE) does not form a ternary complex with She3p and E3-118-tRNA. In size-exclusion chromatography the co-complex of She2p (ΔhE) and E3-118-tRNA (chromatogram 2) eluted at a similar volume as She3p alone (chromatogram 3). When all three components were analyzed together, co-migration but no ternary complex at higher molecular weight was observed (chromatogram 1). Corresponding fractions were analyzed by SDS-PAGE and agarose-gel electrophoresis and are shown below each chromatogram. Dotted lines indicate the peak retention volumes of the individual components. (D) In EMSAs, She2p mutants showed defects in complex formation with *EAR1*-zip-code RNA and She3p. Deletion of the She2p C-terminus impaired but did not abolish RNA-dependent complex assembly with She3p. In contrast, She2p (ΔhE) failed to assemble co-complexes at concentrations up to 250 nM. (E) She2p (ΔC) and She2p (ΔhE) showed impaired RNA binding. RNA filter-binding experiments demonstrated a significantly reduced affinity of both She2p mutants to the four *ASH1* zip codes. Binding of both She2p mutants to the *EAR1* zip code and *WSC2N* was only moderately reduced. However, the She2p (ΔhE) interaction with the *WSC2N* element was almost abolished. In contrast, binding of both She2p mutants to unrelated stem-loop RNAs was not reduced. This suggests that helix E and the C-terminus of She2p are dispensable for unspecific RNA binding. “% wt-binding” indicates the percentage of RNA-binding affinity relative to wild-type She2p binding. Although wild-type She2p data have been published previously [33], these values were obtained from the same series of experiments.
(0.33 MB PDF)

Figure S10 Analysis of She2p point mutants. (A–B) Mutant proteins She2p (E183A D184A G185A) (A) and She2p (T191A D192A) (B) do not show impaired complex formation with *ASH1*-E3-zip-code RNA and She3p in EMSAs. (C) In pull-down experiments, the helix E–affecting mutants She2p (Q197A E198A I199A) and She2p (F195A L196A) failed to interact with immobilized His-She3p (compare lanes 6 and 9 with lane 3; I, input; W, final wash; E, elution). Please note that She2p (F195A L196A) migrated slightly slower than the wild type in SDS-PAGE. However, we confirmed the expected mass of both point mutants by mass spectrometry (unpublished data). (D) Control pull-down experiments showed that none of the two She2p mutants bound to nickel-sepharose beads. (E) RNA filter-binding assays demonstrated a comparably mild, 2.5- to 3-fold reduction in binding of the mutants She2p (Q197A E198A I199A) and She2p (F195A L196A) to the *ASH1*-E3 zip-code RNA. Thus, in these mutants RNA binding seems less severely affected than She3p binding (C). Numbers given in columns “% wt-binding” show the percentage of the RNA-binding affinity relative to wild-type She2p binding, which was determined in a parallel experiment. (F) Circular dichroism spectroscopy with She2p (Q197A E198A I199A), She2p (F195A L196A), and wild-type She2p indicated that none of the mutations impairs protein folding.
(0.42 MB PDF)

Figure S11 Supporting information for chromatin immunoprecipitation (ChIP) experiments. (A) Data of ChIP experiments with anti-TAP antibody in TAP-She2p expressing yeast cells. Upper table shows results comparing TAP-She2p expressing wild-type cells with TAP-She2p expressing Δ*she3* cells. The lower table shows the same experiment with an additional RNase treatment. For further details, see Figure 8B,C (TSS, transcription start site; ORF, open reading frame; pA, polyadenylation site). (B) Western blot with anti-TAP antibody as well as with anti-She2p antibody confirmed that She2p-TAP was expressed at equal levels in wild-type and Δ*she3* cells.
(0.09 MB PDF)

Figure S12 Surface representation of the She2p tetramer [33]. Structure is depicted from the front (left) and rotated by about 90° around the vertical axis (right). The RNA binding basic-helical hairpins (blue) [19] and the protruding helices E with neighboring residues required for She3p and RNA binding (red; this study) are highlighted. The positions of the C-terminal tails, which were absent in the previously published crystal structure [19], are indicated by arrowheads. The indicated distance shows the dimension of the flat RNA-binding surfaces on both sides of the tetramer that are confined by the protruding helices E. The protruding helix E and adjacent amino acids are required for the interaction with She3p and zip-code RNAs (Table 1), for the formation of specific ternary complexes in vitro (Table 1) and in vivo (Figure 7A), as well as for mRNP localization to the bud tip (Figure 7B–E). The functional importance of this surface feature correlates well with its exposed position in the tetrameric structure of She2p [33]. In addition, the flexible C-terminus of She2p is in a position where it could contribute to mRNP stabilization. Size-exclusion chromatography experiments also suggest a slightly disturbed oligomerization of She2p (ΔC) (Figure S9A). For She3p interaction, however, the She2p C-terminus is not required (Figure 6A).
(0.63 MB PDF)

Figure S13 Sequence alignment of She3p from different yeast species. (A) Phylogenetic tree with bootstrap analysis. She3p from *S. cerevisiae* is boxed in yellow. Branches to species without clear She2p homologs are highlighted in red. Numbers at nodes show bootstrap values. (B) Sequence alignment of She3p homologs from species shown in the phylogenetic tree (A). Among all species, the N-terminal, motor-interacting half of She3p shows higher sequence conservation than the C-terminal half, which interacts with She2p in *S. cerevisiae*. Species boxed in red lack clear She2p homologs in their genomes. Red bar with the label “crosslink” highlights amino acids of She3p that were contained in a UV cross-linked peptide of She3p (334–425) (for further details, see Figures 4F and S9A,B). Sequence alignment was performed via Fungal Genome Search using WU-BLAST2 (www.yeastgenome.org). Based on this alignment, the phylogenetic tree (Neighbor Joining algorithm) and alignment representation were prepared using the CLC Sequence Viewer (www.clcbio.com).
(0.61 MB PDF)

Table S1 Plasmids used in this study.
(0.10 MB PDF)

Table S2 Oligonucleotides used in this study.
(0.06 MB PDF)

Table S3 Yeast strains used in this study.
(0.07 MB PDF)

Table S4 Sequences of RNAs used in this study.
(0.09 MB PDF)

Table S5 Primers used for ChIP experiments.
(0.06 MB PDF)

Text S1 Cloning strategy and generation of yeast strains; Circular dichroism spectroscopy; UV cross-linking and enrichment of cross-links for mass spectrometric (MS) analysis; and Nano-LC-ESI-MS analysis of enriched UV cross-links and MS data analysis.
(0.13 MB PDF)

## Abbreviations

ChIP: chromatin immunoprecipitation
EMSA: electrophoretic mobility shift assay
IP: immunoprecipitated
mRNP: messenger ribonucleoprotein particle
ORF: open reading frame
