# Supplementary material for: A Cytoplasmic Complex Mediates Specific mRNA Recognition and Localization in Yeast
Source: PLoS Biol. 2011 Apr 19;9(4):e1000611. doi: 10.1371/journal.pbio.1000611 (PMC3079584; doi:10.1371/journal.pbio.1000611)

Müller et al.

Figure S1

A

Size-exclusion chromatography with Puf6p

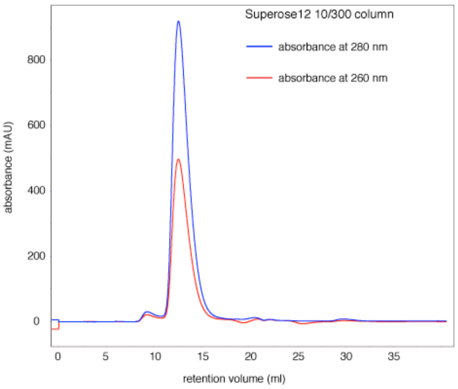

B

Circular dichroism spectroscopy with Puf6p

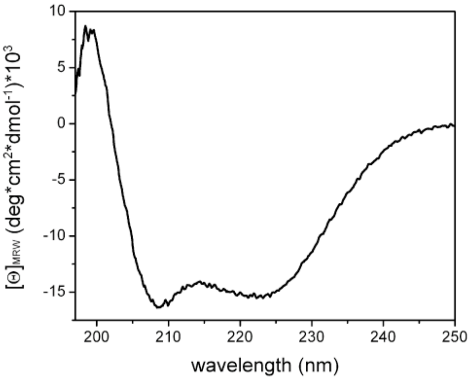

Supplement: Figure S1 — Control experiments to confirm that Puf6p is an intact protein. (A) In size-exclusion chromatography full-length Puf6p eluted as a single, defined peak. No major elution was observed at the void volume of about 8 ml, confirming that Puf6p does not aggregate. The sharp elution peak of Puf6p further indicates a defined conformation of this protein. (B) Circular dichroism spectroscopy with Puf6p reveals a profile typical for alpha-helical proteins. Since Puf6p contains seven Pumilio-homology domains [29], which are highly alpha-helical [51], this profile is expected for correctly folded Puf6p. (0.09 MB PDF) [file pbio.1000611.s001.pdf]
