# Supplementary figures and images for: A Cytoplasmic Complex Mediates Specific mRNA Recognition and Localization in Yeast
Source: PLoS Biol. 2011 Apr 19;9(4):e1000611. doi: 10.1371/journal.pbio.1000611 (PMC3079584; doi:10.1371/journal.pbio.1000611)

Figure S2

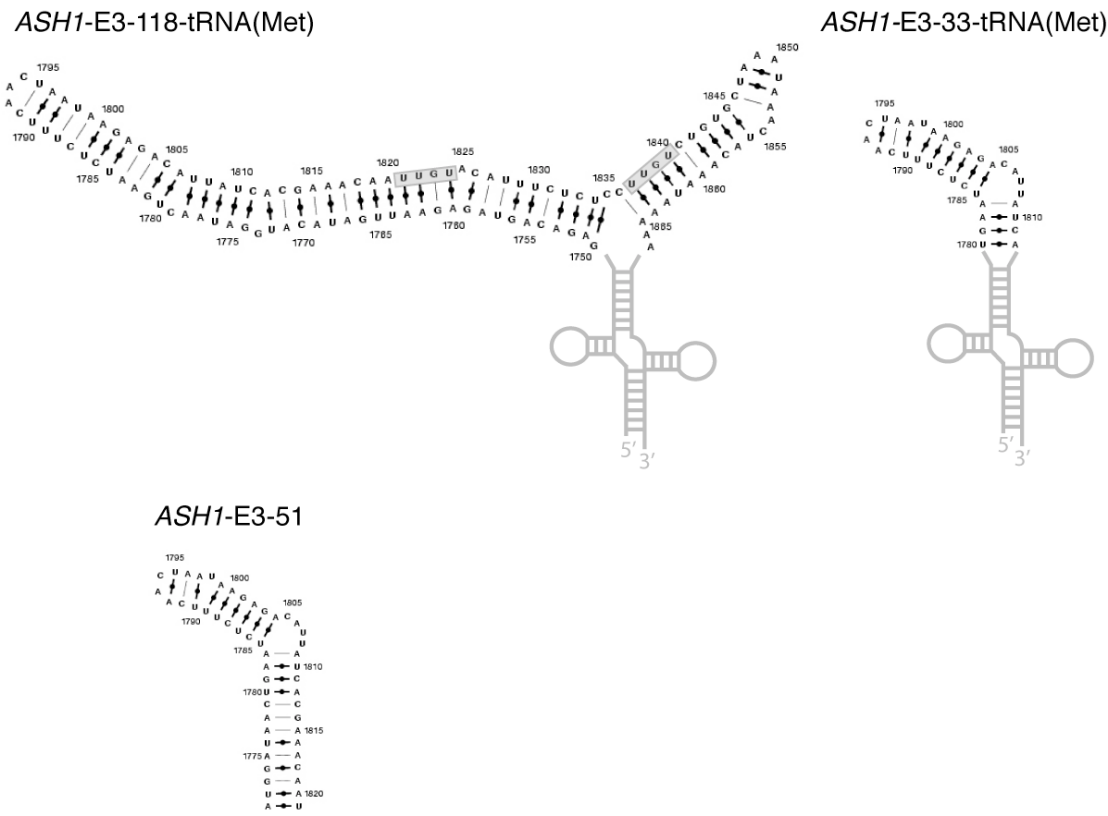

Supplement: Figure S2 — Structure predictions of the ASH1 mRNA variants E3-118, E3-51, and E3-33. Predictions were generated with MC-Fold [52]. Bold lines with dot represent Watson-Crick base pairs, whereas lines indicate non-Watson-Crick base pairs. Grey boxes in E3-118 show the location of the two PUF-consensus sequences UUGU [29]. The numbering starts at base one of the ASH1 mRNA start codon. The zip-code stem loops E3-118 and E3-33 were fused to the anticodon stem of tRNA(Met) [35],[53]. Schemes of tRNAs are not drawn to scale. The complete sequences of the constructs are given in Table S4. (0.16 MB PDF) [file pbio.1000611.s002.pdf]

Figure S3

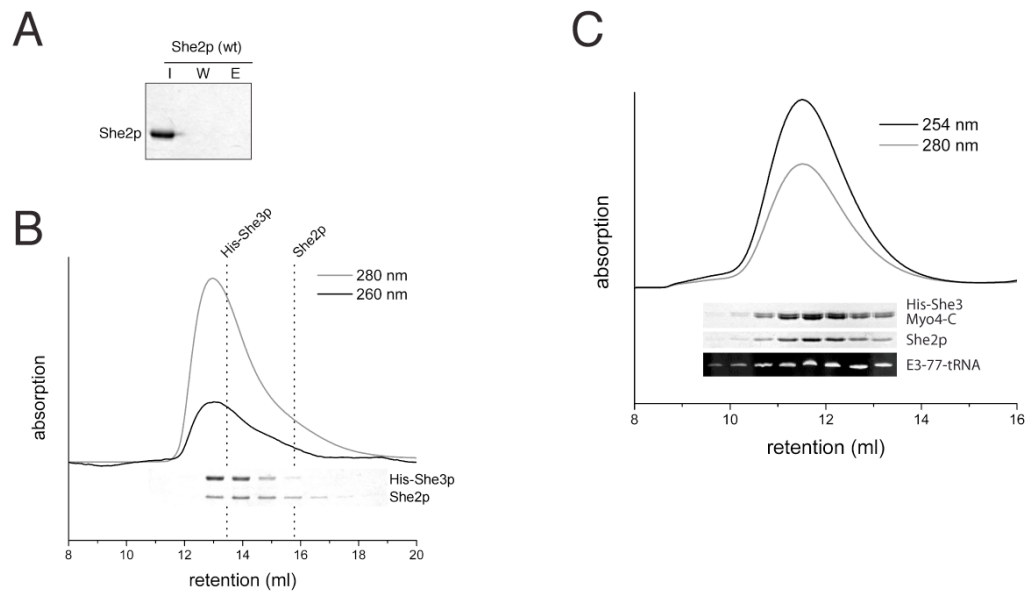

Supplement: Figure S3 — Control experiments for complex assembly with She2p. (A) In pull-down experiments, wild-type She2p did not interact with nickel-sepharose beads. (B) In size-exclusion chromatography experiments, wild-type She2p and She3p eluted as a co-complex in absence of RNA. (C) Size-exclusion chromatography of the reconstituted ASH1-E3 mRNA core complex, consisting of ASH1-E3 RNA, She2p, She3p, and the interacting Myo4p-tail fragment. Corresponding fractions were analyzed by SDS-PAGE and agarose gel electrophoresis and are shown below the chromatogram. (0.15 MB PDF) [file pbio.1000611.s003.pdf]

Figure S4

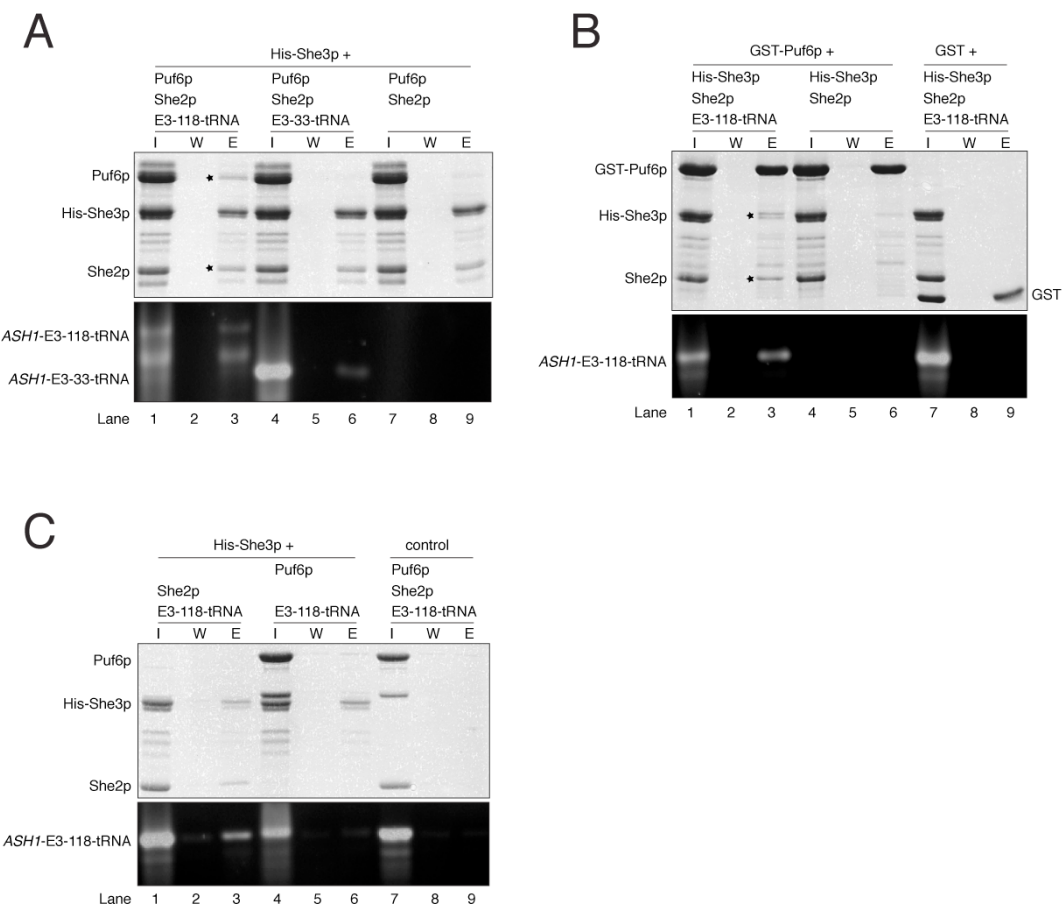

Supplement: Figure S4 — Reconstitution of a major part of the cytoplasmic ASH1-E3 mRNP. (A) In pull-down experiments with immobilized His-She3p, Puf6p and She2p co-eluted in an ASH1-E3 zip-code-dependent manner (lanes 1–3; pulled-down proteins are indicated by asterisks). No co-elution of Puf6p was observed when the control RNA ASH1-E3-33 was used (lanes 4–6) or in absence of RNA (lanes 7–9). As observed previously, She3p bound to She2p also in absence of RNA. The small amount of ASH1-E3-33 RNA eluting with She3p from the nickel sepharose beads (lane 6) most likely resulted from unspecific RNA binding by She3p (see also Figure 3A,B). (B) Pull-down experiments with immobilized GST-tagged Puf6p revealed a complex of Puf6p, She3p, and She2p only in presence of the functional ASH1-E3 zip code (lanes 1–3; pulled-down proteins are marked by an asterisk), but not in absence of RNA (lanes 4–6). Please note that She3p always migrates as a double band in SDS gels. In a control experiment with immobilized GST protein, no unspecific binding of She2p, She3p, or RNA was observed (lanes 7–9). (C) Control pull-down experiments with immobilized His-tagged She3p confirmed the formation of a ternary complex consisting of She3p, She2p, and ASH1-E3 RNA (lanes 1–3) but did not show an interaction of She3p with Puf6p in the presence of ASH1-E3 RNA (lanes 4–6). No unspecific binding of She2p and ASH1-E3 RNA to the beads was observed (lanes 7–9). In contrast, Puf6p retained a weak affinity to nickel sepharose, which could not be eliminated by using 50 mM Imidazole in binding and washing buffers. Please also note that the band migrating above His-She3p represents a degradation product of Puf6p (compare lane 4 with 7). I, input; W, final wash; E, elution. (0.42 MB PDF) [file pbio.1000611.s004.pdf]

Figure S5

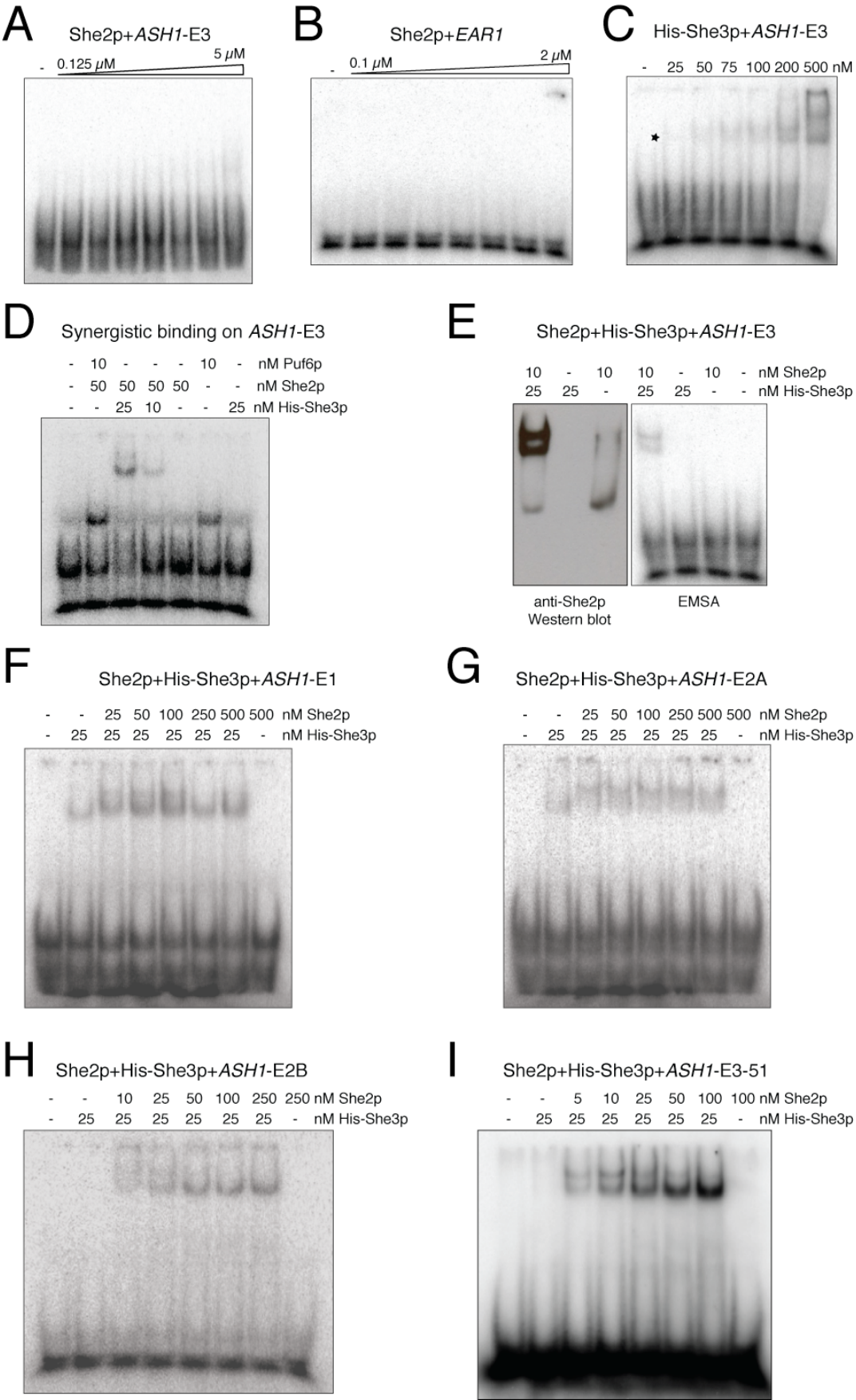

Supplement: Figure S5 — Supporting experiments for studies on synergistic RNA binding by She2p and She3p. (A–B) The She2p:RNA interaction is too transient to be resolved in EMSA experiments. Even at micromolar She2p concentrations, no stable complex with either ASH1-E3 (A) or EAR1 zip-code RNA (B) was detected. (C) The minimal She3p concentration required for detection of ASH1-E3 zip code binding in EMSAs was determined to be in the range of 25 nM to 50 nM (position marked by asterisk). (D) This EMSA shows synergistic binding of She2p and She3p on ASH1-E3 RNA in direct comparison to the unaffected Puf6p binding in presence of She2p. A shift to larger molecular weight and thus the formation of a ternary complex was only observed for She2p and She3p. Puf6p alone showed strong but unspecific binding (see Figure 1). (E) Western blot against She2p after EMSA confirms the presence of She2p in the ternary complex with She3p and ASH1-E3 RNA. (F–I) EMSAs with a constant She3p concentration (25 nM) and varying amounts of She2p show specific ternary complex formation with the ASH1-E1 zip code (F), ASH1-E2A zip code (G), ASH1-E2B zip code (H), and a shortened ASH1-E3-51 zip-code RNA (I). The Kds for these complexes were all estimated to be in a similar range and are comparable to specific complex formation with ASH1-E3 and EAR1 zip codes (see Figure 3C,D). Please note that 25 nM She3p seem to be sufficient for weak binding to ASH1-E1 (F) and ASH1-E2A (G), resulting in the formation of a subtle band shift. However, RNA:She2p:She3p complexes migrated slightly slower in EMSAs and could thus be distinguished from RNA:She3p complexes. (1.11 MB PDF) [file pbio.1000611.s005.pdf]

Figure S6

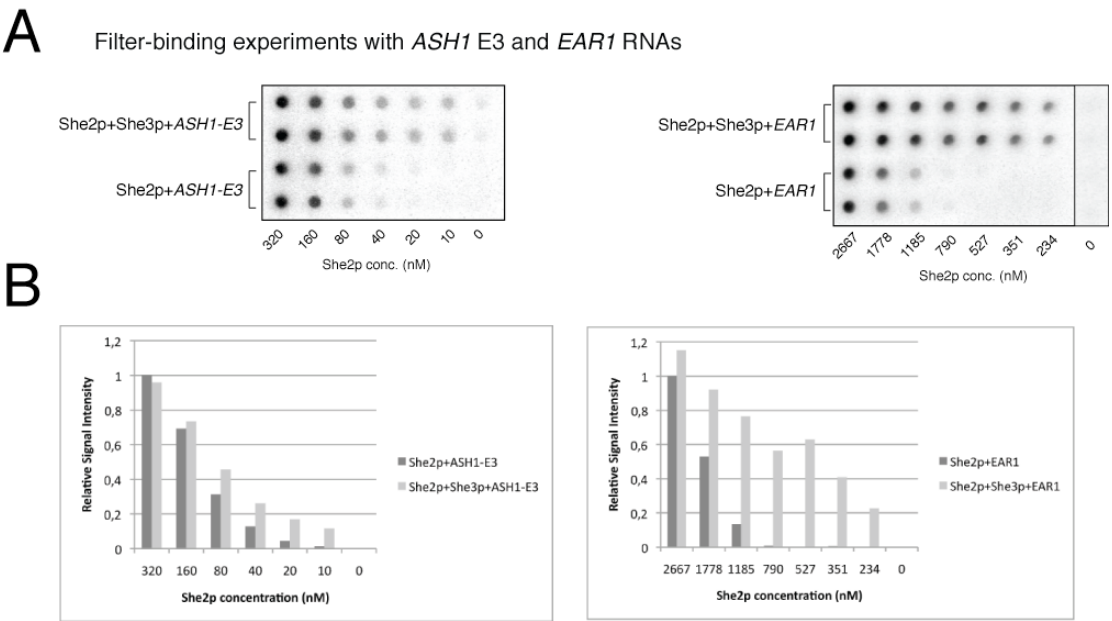

Supplement: Figure S6 — Control experiments for the observed synergistic complex formation of She2p and She3p on zip code containing RNA. (A) Filter-binding experiments confirm the synergistic binding of She2p and She3p to the ASH1-E3 (left) and the EAR1 (right) zip codes. Shown are the raw data obtained by dot-blot experiments with radiolabeled zip-code RNA, various She2p concentrations, and 25 nM She3p whenever indicated. The presence of She3p in the reaction significantly enhanced RNA binding, as determined by an increase in signal intensity at lower She2p concentrations. (B) In the corresponding graphs, relative signal intensities are plotted against the She2p concentrations to visualize the larger amount of zip-code RNA bound by the She2p:She3p complex. Because ternary complex formation does not appear to follow simple reaction kinetics, we refrained from calculating Kd values. (0.19 MB PDF) [file pbio.1000611.s006.pdf]

Figure S7

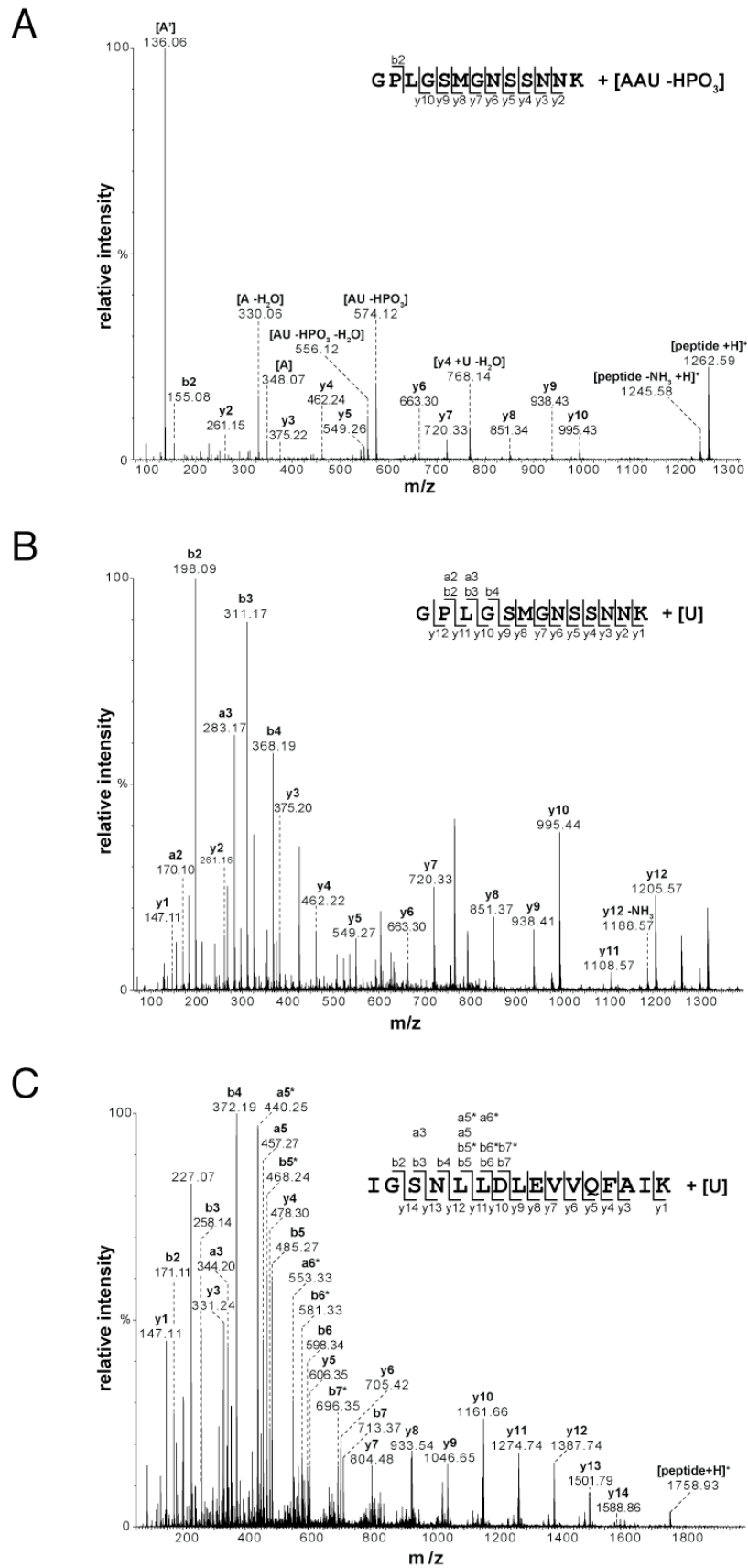

Supplement: Figure S7 — UV cross-linking and subsequent mass spectrometric analysis of RNA-bound protein fragments. The peptide sequence and cross-linked nucleotides as well as identified peptide CID fragments are indicated above each MSMS spectrum. (A) MSMS spectrum of the N-terminal tryptic peptide GPLGSMGNSSNNK (G334–K340) of His-She3 (334–425), including the preceding linker region GPLGSM (underlined), cross-linked to an AAU oligonucleotide without any terminal phosphate. [A] represents the adenosine nucleotide, while [A′] stands for the adenine base. All further RNA-related signals are labeled accordingly. The signal at m/z 768.14 indicates that S10 could be cross-linked to U, as it can be calculated as the y4 peptide fragment with an additional U nucleotide and a loss of H2O. The experimental precursor mass 2,163.68 Da equals the sum of calculated peptide and oligonucleotide masses, 1,261.57 Da and 902.17 Da, respectively, within the mass accuracy of the employed Q-ToF instrument. (B) MSMS spectrum of the N-terminal tryptic peptide GPLGSMGNSSNNK (G334–K340) of His-She3 (334–425) cross-linked to a uridine nucleotide. The peptide's N-terminus is carbamylated due to hydrolyzation in urea at elevated temperatures. The sum of the calculated peptide mass (1,304.58 Da) and the mass of a uridine nucleotide (324.04 Da) equals the experimental precursor mass of 1,628.73 Da. (C) MSMS spectrum of the She2 peptide IGSNLLDLEVVQFAIK (I164–K179) cross-linked to a uridine nucleotide. Peptide fragments with neutral loss of ammonia are marked with an asterisk. The experimental precursor mass of 2,081.92 Da equals the sum of calculated peptide and nucleotide mass (1,757.99 Da and 324.04 Da, respectively). (0.17 MB PDF) [file pbio.1000611.s007.pdf]

Figure S8

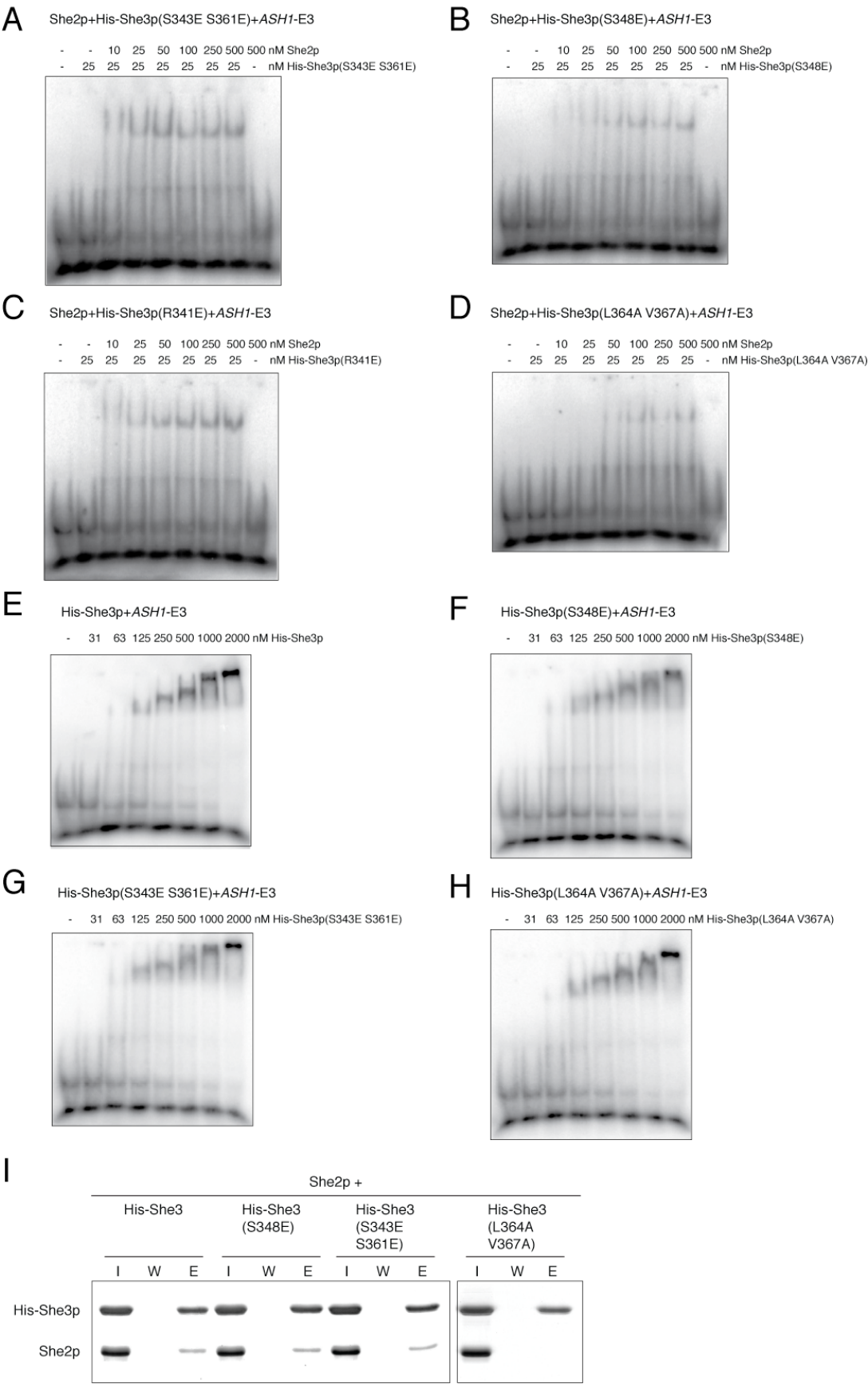

Supplement: Figure S8 — Analysis of She3p point mutants. (A–D) Synergistic binding of His-She3p mutants and wild-type She2p to ASH1-E3 RNA was analyzed by EMSAs. Synergistic RNA binding was not significantly reduced for She3p (S343E S361E) (A) and She3p (R341E) (C) as compared to the wild-type (Figure 3C). She3p (R348E) (B) showed a slightly reduced and She3p (L364A V367A) (D) a strongly reduced synergistic RNA binding. (E–H) Binary interactions of His-She3p mutants with ASH1-E3 RNA were analyzed by EMSAs. RNA binding of She3p (R348E) (F), She3p (S343E S361E) (G), and She3p (L364A V367A) (H) was comparable to wild-type She3p (E). (I) Interactions between His-tagged She3p mutants and wild-type She2p were analyzed by in vitro pull-down with nickel sepharose. She2p binding to She3p (S348E) and She3p (S343E S361E) was comparable to wild-type She3p. In contrast, She3p (L364A V367A) did not interact with She2p. (0.79 MB PDF) [file pbio.1000611.s008.pdf]

Figure S9

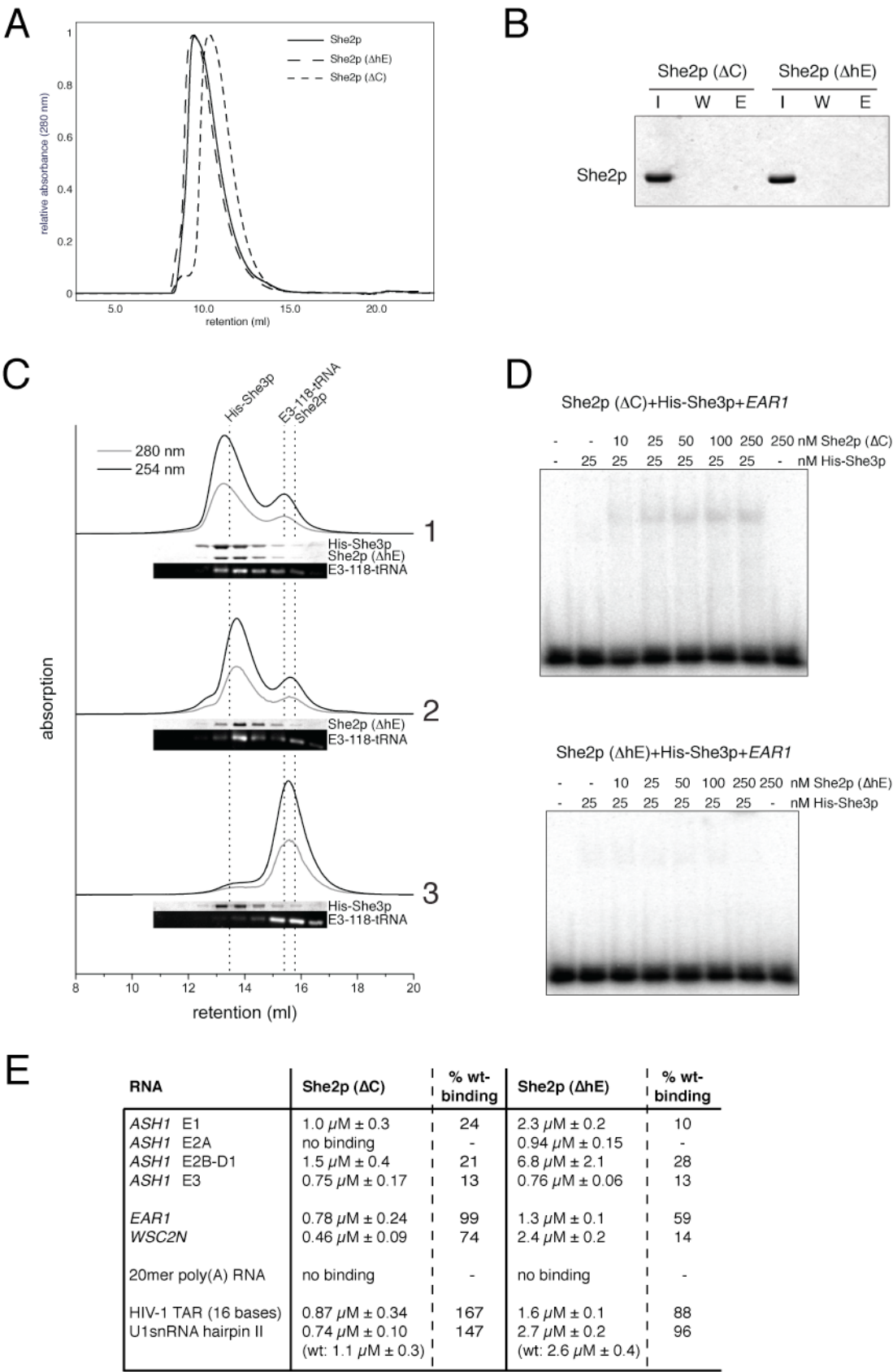

Supplement: Figure S9 — The effect of She2p mutations on RNA binding and complex formation. (A) Elution profiles from preparative size-exclusion chromatography (Superose 12 column) show that wild-type She2p and She2p (ΔhE) eluted at the same volume, whereas She2p (ΔC) showed a retarded elution. Thus, deletion of the C-terminus appears to slightly affect She2p tetramerization without completely disrupting it (for further details, see [33]). The sharp peaks of all She2p fragments confirm protein integrity. (B) Control pull-down experiments show that neither She2p (ΔC) nor She2p (ΔhE) bound to nickel-sepharose beads. (C) She2p (ΔhE) does not form a ternary complex with She3p and E3-118-tRNA. In size-exclusion chromatography the co-complex of She2p (ΔhE) and E3-118-tRNA (chromatogram 2) eluted at a similar volume as She3p alone (chromatogram 3). When all three components were analyzed together, co-migration but no ternary complex at higher molecular weight was observed (chromatogram 1). Corresponding fractions were analyzed by SDS-PAGE and agarose-gel electrophoresis and are shown below each chromatogram. Dotted lines indicate the peak retention volumes of the individual components. (D) In EMSAs, She2p mutants showed defects in complex formation with EAR1-zip-code RNA and She3p. Deletion of the She2p C-terminus impaired but did not abolish RNA-dependent complex assembly with She3p. In contrast, She2p (ΔhE) failed to assemble co-complexes at concentrations up to 250 nM. (E) She2p (ΔC) and She2p (ΔhE) showed impaired RNA binding. RNA filter-binding experiments demonstrated a significantly reduced affinity of both She2p mutants to the four ASH1 zip codes. Binding of both She2p mutants to the EAR1 zip code and WSC2N was only moderately reduced. However, the She2p (ΔhE) interaction with the WSC2N element was almost abolished. In contrast, binding of both She2p mutants to unrelated stem-loop RNAs was not reduced. This suggests that helix E and the C-terminus of She2p are dispensable for unspecific R [file pbio.1000611.s009.pdf]

Figure S10

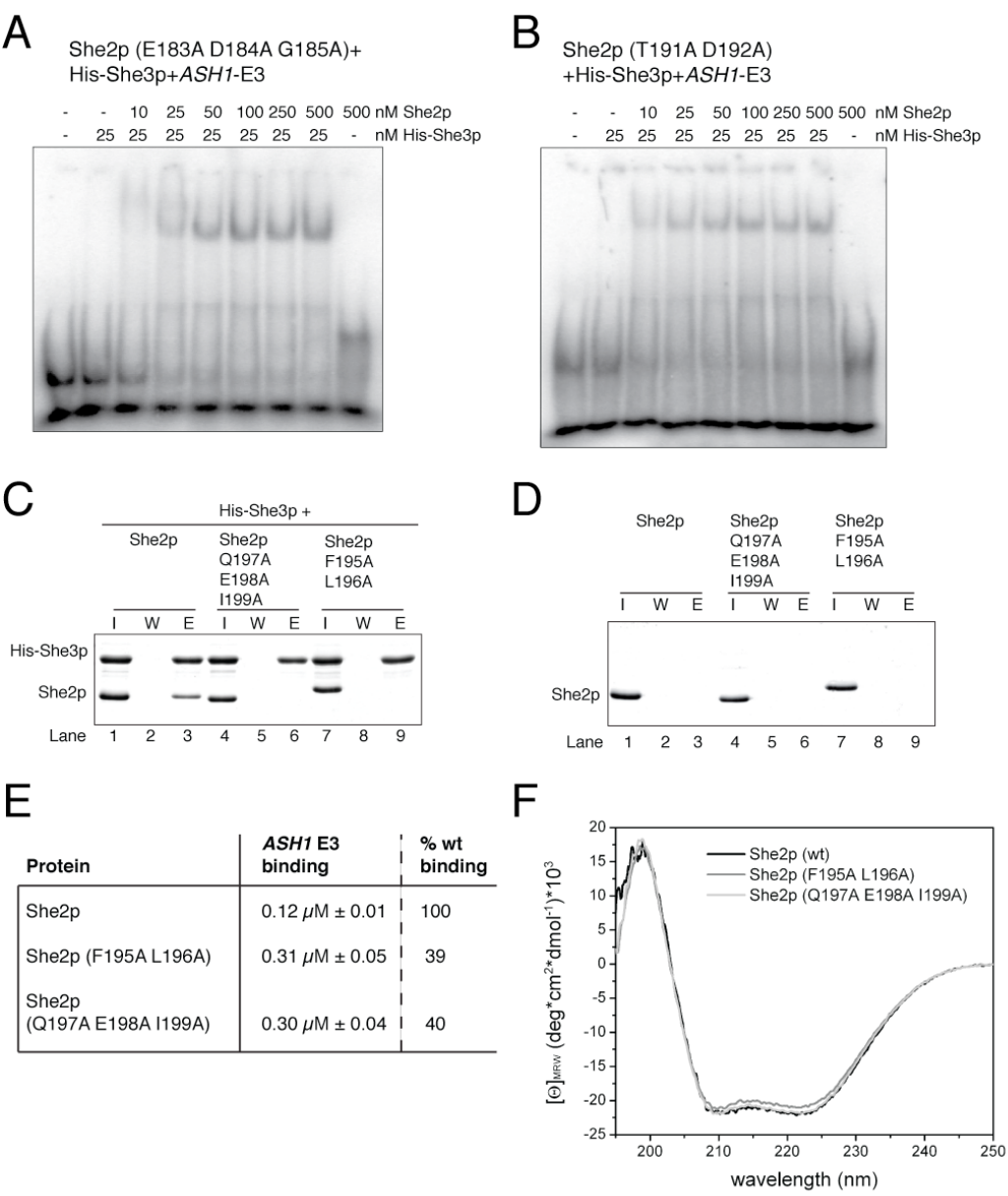

Supplement: Figure S10 — Analysis of She2p point mutants. (A–B) Mutant proteins She2p (E183A D184A G185A) (A) and She2p (T191A D192A) (B) do not show impaired complex formation with ASH1-E3-zip-code RNA and She3p in EMSAs. (C) In pull-down experiments, the helix E–affecting mutants She2p (Q197A E198A I199A) and She2p (F195A L196A) failed to interact with immobilized His-She3p (compare lanes 6 and 9 with lane 3; I, input; W, final wash; E, elution). Please note that She2p (F195A L196A) migrated slightly slower than the wild type in SDS-PAGE. However, we confirmed the expected mass of both point mutants by mass spectrometry (unpublished data). (D) Control pull-down experiments showed that none of the two She2p mutants bound to nickel-sepharose beads. (E) RNA filter-binding assays demonstrated a comparably mild, 2.5- to 3-fold reduction in binding of the mutants She2p (Q197A E198A I199A) and She2p (F195A L196A) to the ASH1-E3 zip-code RNA. Thus, in these mutants RNA binding seems less severely affected than She3p binding (C). Numbers given in columns “% wt-binding” show the percentage of the RNA-binding affinity relative to wild-type She2p binding, which was determined in a parallel experiment. (F) Circular dichroism spectroscopy with She2p (Q197A E198A I199A), She2p (F195A L196A), and wild-type She2p indicated that none of the mutations impairs protein folding. (0.42 MB PDF) [file pbio.1000611.s010.pdf]

Figure S12

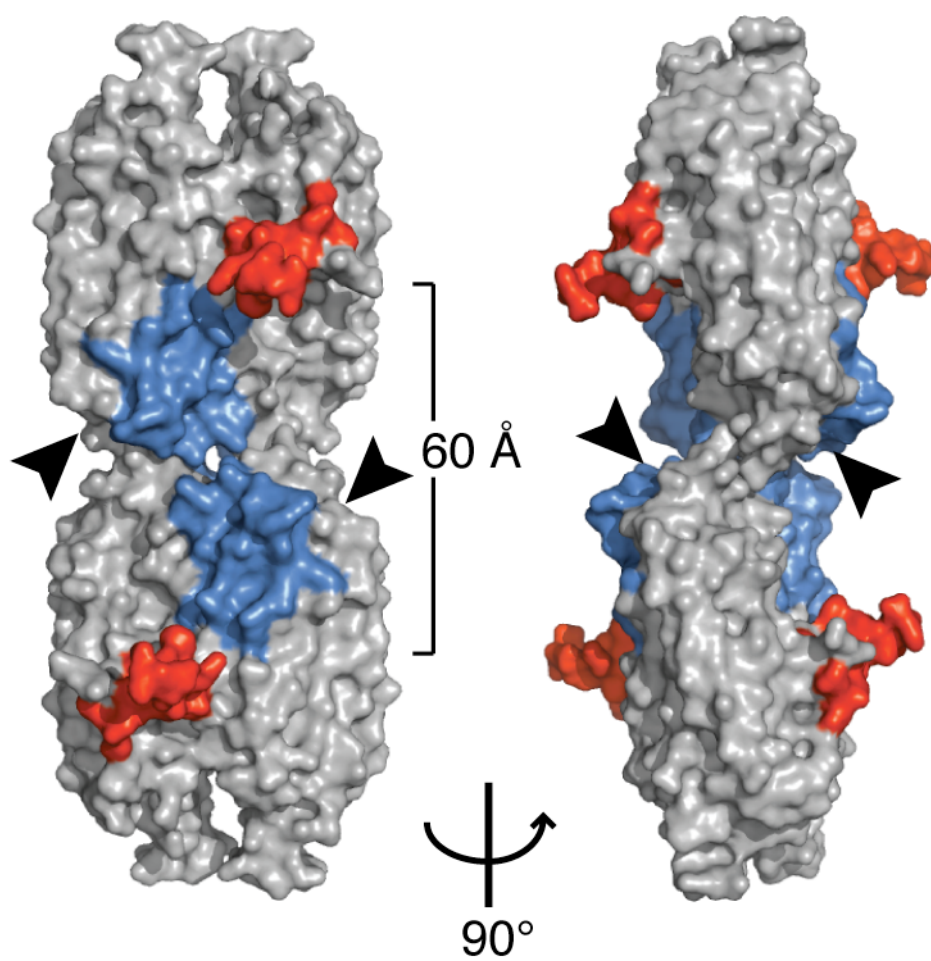

Supplement: Figure S12 — Surface representation of the She2p tetramer [33]. Structure is depicted from the front (left) and rotated by about 90° around the vertical axis (right). The RNA binding basic-helical hairpins (blue) [19] and the protruding helices E with neighboring residues required for She3p and RNA binding (red; this study) are highlighted. The positions of the C-terminal tails, which were absent in the previously published crystal structure [19], are indicated by arrowheads. The indicated distance shows the dimension of the flat RNA-binding surfaces on both sides of the tetramer that are confined by the protruding helices E. The protruding helix E and adjacent amino acids are required for the interaction with She3p and zip-code RNAs (Table 1), for the formation of specific ternary complexes in vitro (Table 1) and in vivo (Figure 7A), as well as for mRNP localization to the bud tip (Figure 7B–E). The functional importance of this surface feature correlates well with its exposed position in the tetrameric structure of She2p [33]. In addition, the flexible C-terminus of She2p is in a position where it could contribute to mRNP stabilization. Size-exclusion chromatography experiments also suggest a slightly disturbed oligomerization of She2p (ΔC) (Figure S9A). For She3p interaction, however, the She2p C-terminus is not required (Figure 6A). (0.63 MB PDF) [file pbio.1000611.s012.pdf]

**Figure S13**

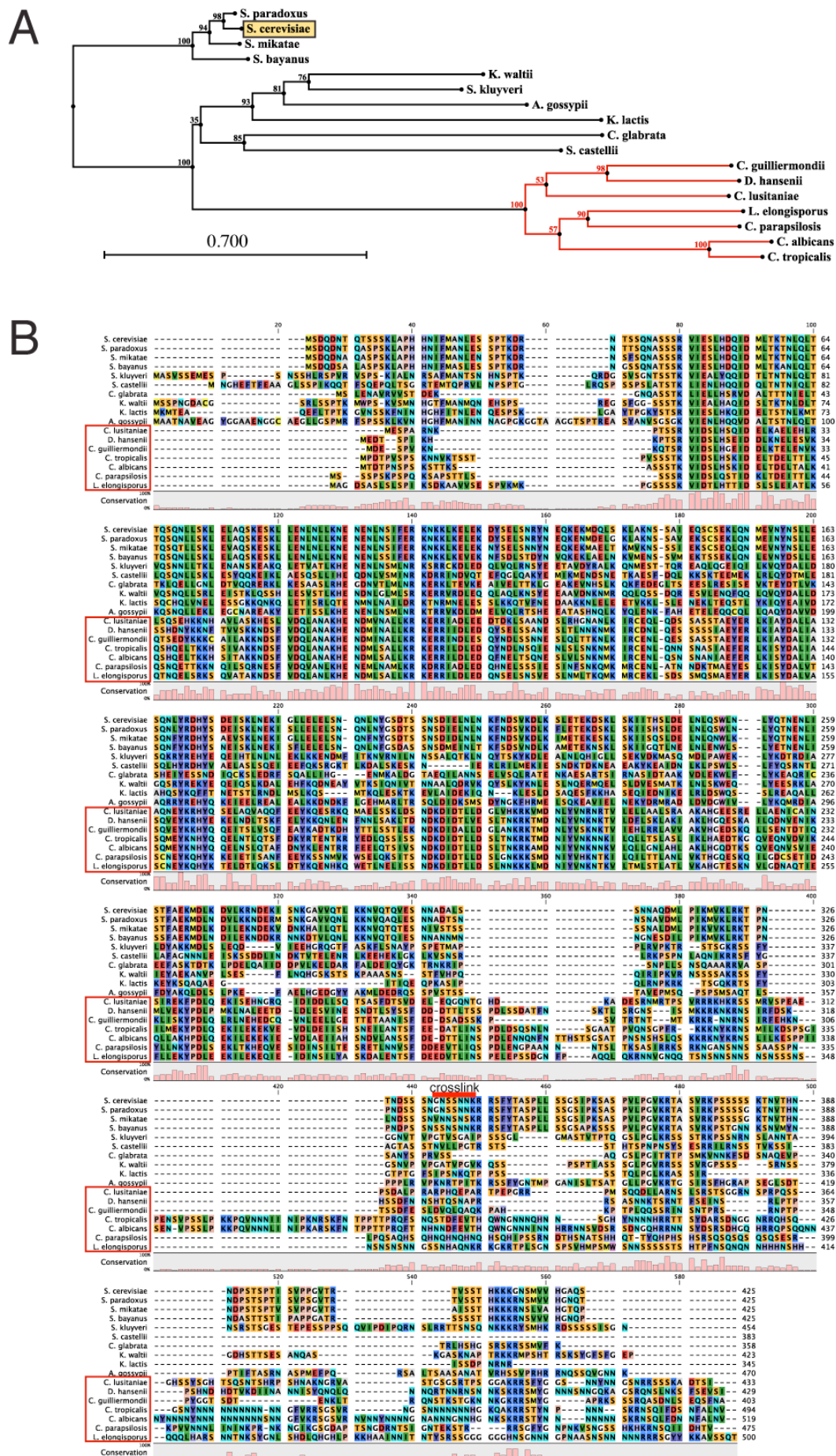

Supplement: Figure S13 — Sequence alignment of She3p from different yeast species. (A) Phylogenetic tree with bootstrap analysis. She3p from S. cerevisiae is boxed in yellow. Branches to species without clear She2p homologs are highlighted in red. Numbers at nodes show bootstrap values. (B) Sequence alignment of She3p homologs from species shown in the phylogenetic tree (A). Among all species, the N-terminal, motor-interacting half of She3p shows higher sequence conservation than the C-terminal half, which interacts with She2p in S. cerevisiae. Species boxed in red lack clear She2p homologs in their genomes. Red bar with the label “crosslink” highlights amino acids of She3p that were contained in a UV cross-linked peptide of She3p (334–425) (for further details, see Figures 4F and S9A,B). Sequence alignment was performed via Fungal Genome Search using WU-BLAST2 (www.yeastgenome.org). Based on this alignment, the phylogenetic tree (Neighbor Joining algorithm) and alignment representation were prepared using the CLC Sequence Viewer (www.clcbio.com). (0.61 MB PDF) [file pbio.1000611.s013.pdf]
