# Supplementary material for: A Cytoplasmic Complex Mediates Specific mRNA Recognition and Localization in Yeast
Source: PLoS Biol. 2011 Apr 19;9(4):e1000611. doi: 10.1371/journal.pbio.1000611 (PMC3079584; doi:10.1371/journal.pbio.1000611)

Müller et al.

Figure S11

A

Chromatin immunoprecipitation experiments with TAP-tagged She2p

Without RNase treatment

	Wild type				Δ*she3*			
	#1	#2	Average	Standard deviation	#1	#2	Average	Standard deviation
ASH1 5'	0.94	1.2	1.1	0.18	1.1	1.1	1.1	0
ASH1 Mid	1.7	1.8	1.8	0.07	1.7	1.5	1.6	0.14
ASH1 3'	2.3	2.3	2.3	0	2.8	2.3	2.6	0.35
ADH1 TSS	0.68	0.84	0.76	0.11	0.89	0.68	0.79	0.15
ADH1 ORF	2.3	2.6	2.5	0.21	3.1	2.8	3.0	0.21
ADH1 pA	3.1	2.8	3.0	0.21	3.5	3.8	3.7	0.21
PMA1 ORF	1.5	1.8	1.7	0.21	2.1	1.8	2.0	0.21
FBA1 ORF	2.0	2.5	2.3	0.35	2.7	2.6	2.7	0.07

With RNase treatment

	Wild type				Δ*she3*			
	#1	#2	Average	Standard deviation	#1	#2	Average	Standard deviation
ASH1 5'	1.0	0,97	0,99	0.02	0,9	1,1	1	0.14
ASH1 Mid	1,4	1,5	1,5	0.07	1.0	1,4	1,2	0.28
ASH1 3'	1,7	1,5	1,6	0.14	1,5	1,6	1,6	0.07
ADH1 TSS	0,51	0,51	0,51	0.01	0,59	0,54	0,57	0.05
ADH1 ORF	1,8	1,8	1,8	0.0	1,7	2,1	1,9	0.28
ADH1 pA	2.0	2.0	2.0	0.0	2,1	1,9	2.0	0.14
PMA1 ORF	1,1	1,2	1,2	0.07	0,9	1,5	1,2	0.42
FBA1 ORF	1,5	1,5	1,5	0.0	1,7	2,1	1,9	0.28

B

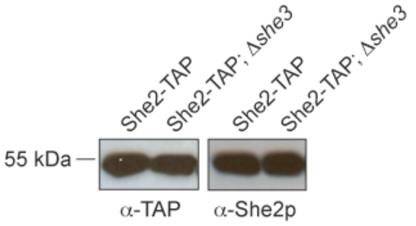

Supplement: Figure S11 — Supporting information for chromatin immunoprecipitation (ChIP) experiments. (A) Data of ChIP experiments with anti-TAP antibody in TAP-She2p expressing yeast cells. Upper table shows results comparing TAP-She2p expressing wild-type cells with TAP-She2p expressing Δshe3 cells. The lower table shows the same experiment with an additional RNase treatment. For further details, see Figure 8B,C (TSS, transcription start site; ORF, open reading frame; pA, polyadenylation site). (B) Western blot with anti-TAP antibody as well as with anti-She2p antibody confirmed that She2p-TAP was expressed at equal levels in wild-type and Δshe3 cells. (0.09 MB PDF) [file pbio.1000611.s011.pdf]
