# Supplementary material for: A Cytoplasmic Complex Mediates Specific mRNA Recognition and Localization in Yeast
Source: PLoS Biol. 2011 Apr 19;9(4):e1000611. doi: 10.1371/journal.pbio.1000611 (PMC3079584; doi:10.1371/journal.pbio.1000611)
Supplement: Table S1 — Plasmids used in this study. (0.10 MB PDF) [file pbio.1000611.s014.pdf]

Table S1

Plasmids used in this study

**Plasmid Name**	**Description**	**Source**
p01	pGEX-*SHE2*	[1]
p06	pGEX-*SHE2-ΔC*	this study
p11	pGEX-*SHE2-ΔhE*	this study
RHP 118	pGEX-*SHE2(E183A D184A G185A)*	this study
RHP 119	pGEX-*SHE2(T191A D192A)*	this study
RHP 138	pGEX-*SHE2(F195A L196A)*	this study
RHP 139	pGEX-*SHE2(Q197A E198A I199A)*	this study
RHP 18	pGEX-*PUF6*	this study
RHP 27	pFastBacDual-*SHE2-SHE3His6*	this study
RHP 113	pFastBacDual-*SHE2-SHE3(S348E)His6*	this study
RHP 114	pFastBacDual-*SHE2-SHE3(S343E S361E)His6*	this study
RHP 129	pFastBacDual-*SHE2-SHE3(L364A V367A)His6*	this study
RHP 130	pFastBacDual-*SHE2-SHE3(R341E)His6*	this study
RHP 73	pGEX-*SHE3(354-425)His6*	this study
RHP 85	pGEX-*SHE3(334-425)His6*	this study
RHP 137	petM40-*SHE3(354-425)His6*	this study
RHP 136	petM40-*SHE3(334-425)His6*	this study
RHP 65	pBSMrna-*ASH1-E3-33*	this study
RHP 60	pBSMrna-*ASH1-E3-77*	this study
RHP 89	pBSMrna-*ASH1-E3-118*	this study
p15	YCplac22-*SHE2-fl-myc3*	[2]
p17	YCplac22-*SHE2-ΔhE-myc3*	this study
p18	YCplac22-*SHE2-ΔC-myc3*	this study
p20	YCplac111-*SHE2-ΔhE*	this study
p21	YCplac111-*SHE2-ΔC*	this study
RJP 132	YEplac195-*ASH1*	[3]
RJP 138	YCplac22	[4]
RJP 145	YCplac111	[4]

RJP 1565	YCplac22-*SHE2-myc3*	[2]
RJP 916	YCplac111-*SHE2*	[5]

References:

1. Niessing D, Hüttelmaier S, Zenklusen D, Singer RH, Burley SK (2004) She2p is a Novel RNA-Binding Protein with a Basic Helical Hairpin Motif. Cell 119: 491-502.
2. Müller M, Richter K, Heuck A, Kremmer E, Buchner J, et al. (2009) Formation of She2p tetramers is required for mRNA binding, mRNP assembly, and localization. RNA 15: 2002-2012.
3. Long RM, Singer RH, Meng X, Gonzalez I, Nasmyth K, et al. (1997) Mating type switching in yeast controlled by asymmetric localization of *ASH1* mRNA. Science 277: 383-387.
4. Gietz RD, Sugino A (1988) New yeast-Escherichia coli shuttle vectors constructed with in vitro mutagenized yeast genes lacking six-base pair restriction sites. Gene 74: 527-534.
5. Du TG, Jellbauer S, Müller M, Schmid M, Niessing D, et al. (2008) Nuclear transit of the RNA-binding protein She2p is required for translational control of localized ASH1 mRNA. EMBO Rep 9: 781-787.
