# Supplementary material for: A Cytoplasmic Complex Mediates Specific mRNA Recognition and Localization in Yeast
Source: PLoS Biol. 2011 Apr 19;9(4):e1000611. doi: 10.1371/journal.pbio.1000611 (PMC3079584; doi:10.1371/journal.pbio.1000611)
Supplement: Table S2 — Oligonucleotides used in this study. (0.06 MB PDF) [file pbio.1000611.s015.pdf]

Table S2

Oligonucleotides used in this study

Primer Name	Primer Sequence (5' - 3')
S2-1	TTACTCGAGTCAGTTTTTCAATTTACCAAATTTG
S2-3	AAAGGATCCATGAGCAAAGACAAAGATATC
S2-4	TCTCCGATATTCGTTCCGTCAACTTCTAAATCTAGCAGGTTGCTGC
S2-5	TGCTAGATTTAGAAGTTGACGGAACGAATATCGGAGAAACTG
S2-17	AAAAAATGCATCAGAGTTTATCAATGAAGG
S2-18	AAAAAAGGCCTCAGTTTTTCAATTTACC
S2-20	AAATCTAGATTTGTCATGCCATTTAGTAGCGAC
S2-21	AAAAAAGGCCTCATTTGTCATGCCATTTAGTAGCGAC
S2-22	TAACTCGAGTTTGTCATGCCATTTAGTAGCGAC
S2-26	GTCTCTAGACAATTTACCAAATTTGTCATGCC
AHO-6	AAAGGATCCATGTCGGACCAGGATAATACC
RHO-25	AAACCCGGGTGCACCTTTAACCAAGAAGAC
RHO-26	TTTCTCGAGTTATTTTAACAATTTAGCCAAAAGCTG
RHO-48	AAACCCGGGATGAGCAAAGACAAAGATATCAAAG
RHO-49	TTTGAATTCCTAGTGATGGTGATGGTGATGGGATTGGGCCCCGTG
RHO-83	GGATCCATGGGTTCTATTCCAAAATCTGCATC
RHO-96	AAACGGCCGAGAGAATTGATACATGGATAACTGAATC
RHO-101	AAACCGCGGCCAAGAGAAATGTACAATTGTTTCGTG
RHO-106	GGCCTGAATCTCTTTCAACTAATAAGAGACATTATCAGGCCGC
RHO-107	GGCCTGATAATGTCTCTTATTAGTTGAAAGAGATTCA
RHO-122	AAAGGATCCATGGGTAATAGCAGTAACAATAAAAG
RHO-151	CTATACTGCGGAGCCTTTGCTGTC
RHO-152	GACAGCAAAGGCTCCGCAGTATAG
RHO-153	GTAACAATAAAAGAAGAGAATTCTATACTGCG
RHO-154	CGCAGTATAGAATTCTCTTCTTTTATTGTTAC
RHO-155	CCAAAATCTGCAGAGCCGGTTTTGCCTGG
RHO-156	CCAGGCAAAACCGGCTCTGCAGATTTTGG
RHO-157	CATTAAATGCGCAGATGCGGCCGCAACGAATATCGG

RHO-158	CCGATATTCGTTGCGGCCGCATCTGCGCATTTAATG
RHO-159	GAATATCGGAGAAGCTGCTAATATCTTCTTGC
RHO-160	GCAAGAAGATATTAGCAGCTTCTCCGATATTC
RHO-162	CATCCCCGGTTGCGCCTGGTGCTAAAAGAACTGC
RHO-163	GCAGTTCTTTTAGCACCAGGCGCAACCGGGGATG
RHO-164	GTAATAGCAGTAACAATAAAGAAAGAAGTTTCTATACTG
RHO-165	CAGTATAGAAACTTCTTTCTTTATTGTTACTGCTATTAC
RHO-171	GAAACTGATAATATCGCCGCGCAGGAGATTCTTC
RHO-172	GAAGAATCTCCTGCGCGGCGATATTATCAGTTTC
RHO-173	CTGATAATATCTTCTTGGCGGCGGCTCTTCCTGTCAACTC
RHO-174	GAGTTGACAGGAAGAGCCGCCGCCAAGAAGATATTATCAG
SC-5	AAACGGCCGGAGACAGTAGAGAATTGATAC
SC-6	AAACCGCGGTTTTTATTTGTAGTTTATTTAGC
