# Supplementary material for: A Cytoplasmic Complex Mediates Specific mRNA Recognition and Localization in Yeast
Source: PLoS Biol. 2011 Apr 19;9(4):e1000611. doi: 10.1371/journal.pbio.1000611 (PMC3079584; doi:10.1371/journal.pbio.1000611)
Supplement: Table S3 — Yeast strains used in this study. (0.07 MB PDF) [file pbio.1000611.s016.pdf]

Müller et al.

Table S3

Yeast strains used in this study

**Strain**	**Essential genotype**
RJY 2053	*MAT alpha; his3Δ1;leu2Δ0, lys2Δ0, ura3Δ0; she2::kanMX4*
y01	*MAT alpha; his3Δ1;leu2Δ0, lys2Δ0, ura3Δ0; she2::kanMX4, YEplac195-ASH1 (RJP132), YCplac111 (RJP145)*
y02	*MAT alpha; his3Δ1;leu2Δ0, lys2Δ0, ura3Δ0; she2::kanMX4, YEplac195-ASH1 (RJP132), YCplac111-SHE2 (RJP916)*
y03	*MAT alpha; his3Δ1;leu2Δ0, lys2Δ0, ura3Δ0; she2::kanMX4, YEplac195-ASH1 (RJP132), YCplac111-SHE2-ΔhE (p20)*
y04	*MAT alpha; his3Δ1;leu2Δ0, lys2Δ0, ura3Δ0; she2::kanMX4, YEplac195-ASH1 (RJP132), YCplac111-SHE2-ΔC (p21)*
RJY 3364	*MAT a, his3, leu2, ade2, trp1, ura3, HO-ADE2, HO-CAN1, HA3-SHE3, she2::natNT2*
y06	*MAT a, his3, leu2, ade2, trp1, ura3, HO-ADE2, HO-CAN1, HA3-SHE3, she2::natNT2, YCplac22 (RJP138)*
y07	*MAT a, his3, leu2, ade2, trp1, ura3, HO-ADE2, HO-CAN1, HA3-SHE3, she2::natNT2, YCplac22-SHE2-myc3(p15)*
y08	*MAT a, his3, leu2, ade2, trp1, ura3, HO-ADE2, HO-CAN1, HA3-SHE3, she2::natNT2, YCplac22-SHE2-ΔhE-myc3 (p17)*
y09	*MAT a, his3, leu2, ade2, trp1, ura3, HO-ADE2, HO-CAN1, HA3-SHE3, she2::natNT2, YCplac22-SHE2-ΔC-myc3 (p18)*
RJY1543	*MAT alpha, trp1-1, leu2-3, his3-11, ura3-52, mex67::S.p.HIS3, SHE3-HA6::K.l.TRP1, SHE2-myc3::S.p.HIS3, pUN100-mex67-5*
