# Supplementary material for: A Cytoplasmic Complex Mediates Specific mRNA Recognition and Localization in Yeast
Source: PLoS Biol. 2011 Apr 19;9(4):e1000611. doi: 10.1371/journal.pbio.1000611 (PMC3079584; doi:10.1371/journal.pbio.1000611)
Supplement: Table S4 — Sequences of RNAs used in this study. (0.09 MB PDF) [file pbio.1000611.s017.pdf]

Table S4:

Sequences of RNAs used in this study

RNA	Base no. relative to start codon	Sequence (5'-3')	Produced by
ASH1 E1	623-701	AAGACUAUGUUAAAAUACGCGAAGAAGUGGCUCAUUUCAAGCCAUUAAGUAUACCCAACUUAACUAAUAAUCAAAAUAA	*in vitro* transcription
ASH1 E2A	1109-1185	UUGCGAAUAGAGACAUUCUAUCGAACAAUUCCAAAUCUAAUGUAAGGAAACCAUCUAAGAACAAAAUCUCAAAGCAA	*in vitro* transcription
ASH1 E2B (domain 1)	1263-1320	AUCUUCAUCUCCAUCUCCCUCCACACCGACGAAAAGUGGCAAGAUGAGAUCAAGAUCA	*in vitro* transcription
ASH1 E3-118	1750-1867	GAGACAGUAGAGAAUUGAUACAUGGAUAACUGAAUCUCUUUCAACUAAUAAGAGACAUUAUCACGAAACAAUUGUACAUUUCUCUCCUUGUCUGUGCUAAAUAAACUACAAAUAAAAA	*in vitro* transcription
ASH1 E3-51	1771-1821	AUGGAUAACUGAAUCUCUUUCAACUAAUAAGAGACAUUAUCACGAAACAAU	chemical synthesis (Dharmacon)
EAR1	1572-1621	CGAAGAUGAAAAUGAUCACGAGAGAGAUCCCGAACAAUUUUCAGAAUUUG	chemical synthesis (Dharmacon); 5' biotinylated
WSC2N	430-465	AGUUCAAAAACGUCCACGAAAUUGGACACGAAAACU	chemical synthesis (Dharmacon); 5' biotinylated
HIV-1 TAR (16 bases)		CGAGCCUGGGAGCUCG	chemical synthesis (Dharmacon)
HIV-1 TAR (57 bases)		GGUCUCUCUGGUUAGACCAGAUCUGAGCCUGGGAGCUCUCUGGCUAACUAGGGAACC	*in vitro* transcription
U1snRNA hairpin II		UUAUCCAUUGCACUCCGGAUGU	chemical synthesis (Dharmacon)
Poly(A)$_{20}$		AAAAAAAAAAAAAAAAAAAA	*in vitro* transcription
Poly(A)$_{60}$		AAAAAAAAAAAAAAAAAAAAAAAAAAAAAAAAAAAAAAAAAAAAAAAAAAAAAAAAAAAA	*in vitro* transcription
ASH1 E3-33-tRNA(Met)*	1780-1812 (underlined sequence)	GGCUACGUAGCUCAGUUGGUUAGAGCAGCGGCCUGAAUCUCUUUCAACUAAUAAGAGACAUUAUCAGGCCGCGGGUCACAGGUUCGAAUCCCGUCGUAGCCACCA	expression and purification from *E. coli*
ASH1 E3-77-tRNA(Met)*	1758-1834	GGCUACGUAGCUCAGUUGGUUAGAGCAGCGGCCGAGAGAAUUGAUACAUGGAUAACUGAAUCUCUUUCAACUAAUAAGAGACAUUAUCACGAAACAAUUGUACAUUUCUCUUGGCCGCGGGUCACAGGUUCGAAUCCCGUCGUAGCCACCA	expression and purification from *E. coli*
ASH1 E3-118-tRNA(Met)*	1750-1867 (underlined sequence)	GGCUACGUAGCUCAGUUGGUUAGAGCAGCGGCCGGAGACAGUAGAGAAUUGAUACAUGGAUAACUGAAUCUCUUUCAACUAAUAAGAGACAUUAUCACGAAACAAUUGUACAUUUCUCUCCUUGUCUGUGCUAAAUAAACUACAAAUAAAAACCGCGGGUCACAGGUUCGAAUCCCGUCGUAGCCACCA	expression and purification from *E. coli*

* *ASH1* E3 sequences are underlined.
