# Supplementary material for: A Cytoplasmic Complex Mediates Specific mRNA Recognition and Localization in Yeast
Source: PLoS Biol. 2011 Apr 19;9(4):e1000611. doi: 10.1371/journal.pbio.1000611 (PMC3079584; doi:10.1371/journal.pbio.1000611)
Supplement: Table S5 — Primers used for ChIP experiments. (0.06 MB PDF) [file pbio.1000611.s018.pdf]

Table S5

Primers used for ChIP experiments.

Primer	Sequence	Location relative to start ATG
ADH1_TSS_Fw	TCCTTGTTTCTTTTTCTGCAC	-67 to -47
ADH1_TSS_Rev	GAGATAGTTGATTGTATGCTTGG	-30 to -8
ADH1_ORF_Fw	AGCCGCTCACATTCCTCAAG	408 to 427
ADH1_ORF_Rev	ACGGTGATACCAGCACACAAGA	455 to 476
ADH1_pA_Fw	AAAACGAAAATTCTTATTCTTGA	1137 to 1159
ADH1_pA_Rev	TACCTGAGAAAGCAACCTGA	1178 to 1197
ASH1_5'_ORF_Fw	CCAATTCTTTCCCCAATATC	205 to 224
ASH1_5'_ORF_Rev	TGCTGGTGCAGTATTTGATT	251 to 270
ASH1_mid_ORF_Fw	TTCCAACAATGCATGGAGTA	834 to 853
ASH1_mid_ORF_Rev	CATGGTTCTATTGGTTGGTG	884 to 903
ASH1_3'_ORF_Fw	CCCACAAAGGGTGAAATAAA	1636 to 1655
ASH1_3'_ORF_Rev	GGGGACGAATTCTTTGTCTA	1676 to 1695
PMA1_ORF_Fw	AAATCTTGGGTGTTATGCCATGT	1574 to 1596
PMA1_ORF_Rev	CCAAGTGTCTAGCTTCGCTAACAG	1628 to 1651
FBA1_ORF_Fw	CGTCACCTCTTCTTCTACTGC	111 to 131
FBA1_ORF_Rev	CAAAATGATTGGGGACTTG	162 to 180
