# Supplementary material for: A Cytoplasmic Complex Mediates Specific mRNA Recognition and Localization in Yeast
Source: PLoS Biol. 2011 Apr 19;9(4):e1000611. doi: 10.1371/journal.pbio.1000611 (PMC3079584; doi:10.1371/journal.pbio.1000611)
Supplement: Text S1 — Cloning strategy and generation of yeast strains; Circular dichroism spectroscopy; UV cross-linking and enrichment of cross-links for mass spectrometric (MS) analysis; and Nano-LC-ESI-MS analysis of enriched UV cross-links and MS data analysis. (0.13 MB PDF) [file pbio.1000611.s019.pdf]

Cloning strategy. A complete list of plasmids used in this study is shown in Table S1. Sequences of all oligonucleotides are listed in Table S2. All *SHE2* fragments for recombinant protein expression were amplified by PCR using plasmid p01 as template [1] and cloned into the vector pGEX-6P-1 (GE Healthcare) by using BamHI and XhoI sites in the respective 5' and 3' primer sites. Plasmid p06 (pGEX-*SHE2-ΔC*) was created using primer pair S2-3/22. Plasmid p11 (pGEX-*SHE2-ΔhE*) was created by site-directed mutagenesis using primer pairs S2-3/4 and S2-5/1. Plasmids RHP 118, RHP 119, RHP 138, and RHP 139 were created using the QuickChange II XL Site-Directed Mutagenesis Kit (Stratagene) with the primer pairs RHO-157/158, RHO-159/160, RHO-171/172, and RHO173/174. To create plasmid RHP 27, full-length *SHE2* was produced by PCR with the primer pair RHO-48/S2-1 and full-length *SHE3* was amplified from genomic yeast DNA using the primer pair AHO-6/RHO-49, introducing a C-terminal His6-tag. The resulting PCR products were cloned into the pFastBacDual vector (Invitrogen) via SmaI/XhoI (*SHE2*) and BamHI/EcoRI (*SHE3His6*) restriction sites. Plasmids RHP 113, RHP 114, RHP 129, and RHP 130 were created using the QuickChange II XL Site-Directed Mutagenesis Kit (Stratagene) with plasmid RHP27 as template and the primer pairs RHO-151/152, RHO-153/154/155/156, RHO-162/163, and RHO-164/165. *SHE3* fragments for recombinant protein expression in *E. coli* were produced by PCR using RHP 27 as template and the primer pairs RHO-83/49 (*SHE3(354-425)His6*) and RHO-122/49 (*SHE3(334-425)His6*). The PCR products were cloned into the vector pGEX-6P-1 (RHP 73, RHP 85) and into a modified petM40 vector (EMBL) (RHP 136, RHP 137) using BamHI and EcoRI restriction sites. Full-length *PUF6* was amplified from genomic yeast DNA by PCR using the primer pair RHO-25/26. To create plasmid RHP 18, the PCR product was cloned into pGEX-6P-1 via SmaI and XhoI restriction sites.

ASH1 E3-118 (RHP 89) and *ASH1* E3-77 (RHP 60) were amplified by PCR from genomic yeast DNA using the primer pairs SC-5/6 and RHO-96/101. The PCR products were cloned into the vector pBSMrna [2] via EagI and SacII restriction sites. For plasmid RHP 65, the primer pair RHO-106/107 was annealed and directly ligated into pBSMrna via EagI and SacII restriction sites.

Construction of plasmids p15 and RJP 1565 has been previously reported [3]. p18 was created by amplifying *SHE2-ΔC* from p01 using primers S2-17 and S2-20 and following subcloning into RJP 1565 using NsiI/XbaI restriction sites. Accordingly, plasmid p17 was created using plasmid p11 as PCR template and the primer pair S2-

17/26. Construction of plasmid RJP 916 has been reported previously [4]. Plasmids p20 and p21 were created by subcloning respective *SHE2* fragments into RJP 916 as follows: *SHE2-ΔhE* was amplified using oligos S2-17/18 from plasmid p11, and *SHE2-ΔC* was amplified from p06 with primer pair S2-17/21. PCR products were digested with NsiI and StuI and cloned into NsiI/StuI digested RJP 916.

Generation of yeast strains. Handling of yeast strains was performed as described [5,6]. Yeast strains used in this study are listed in Table S3. Generation of yeast strains y01 and y02 based on strain RJY 2053 has been described previously [3]. Accordingly, strains y03 and y04 were derived from RJY 2053 by transformation of plasmid RJP 132 and corresponding plasmids p20 and p21, respectively. Construction of strains RJY 3364, y06, and y07 has already been described [3]. Strains y08 and y09 were generated from RJY 3364 by transformation of corresponding plasmids p17 and p18, respectively.

Circular dichroism spectroscopy. Circula dichroism spectra were measured with a Jasco J-810 spectropolarimeter. Proteins were diluted to 0.2-0.3 mg/ml with 10 mM NaH_2PO_4/Na_2HPO_4 (pH 7.5) supplemented with either 50 mM NaCl (She2p) or 150 mM NaCl (Puf6p). Spectra were recorded at 20°C, averaged and corrected for the buffer.

UV cross-linking and enrichment of cross-links for mass spectrometric (MS) analysis. The *ASH1*-E3-51 RNA was dissolved in 20 mM Hepes (pH 7.4), 200 mM NaCl, 2 mM $MgCl_2$ and refolded by heating to 90°C for 5 minutes followed by cooling to 25°C over 22 minutes. *ASH1*-E3-51 RNA was mixed with purified She2p and His-She3p (334-425) in HNMD-buffer. The ternary complex was then purified by size-exclusion chromatography (Superose 6 10/300 GL) in HNMD-buffer. UV cross-linking and enrichment were performed according to established protocols [7,8]). 100 µg of the ternary complex in a volume of 200 µL were transferred to a black polypropylene microtiterplate (Greiner Bio-One, Frichenhausen, Germany) in 100 µL aliquots and subsequently cross-linked for 10 minutes at 254 nm (cross-linking apparatus built in-house, equipped with four 8 W lamps, F8T5BL, Sankyo Denki, Japan). After ethanol precipitation, the sample was reconstituted in the presence of 1 M urea and 50 mM Tris pH 7.9. The sample was incubated with 2 µg RNase A and 2 U RNase T1 (both Ambion, Applied Biosystems, Darmstadt, Germany) for 2 h at 52°C, followed by proteolysis with trypsin (protein:enzyme 20:1, w/w; Promega,

Mannheim, Germany) at 37°C over night. The sample was desalted on a C18 column and cross-linked peptides were subsequently enriched on a TiO_2 column (columns prepared in-house; C18 material: Dr. Maisch GmbH, Ammerbuch, Germany; TiO_2 material: FL Sciences, Tokyo, Japan). The enrichment procedure was performed according to the protocol described in [9]: The sample was dissolved in 200 mg/mL 2,5-dihydroxy benzoic acid (DHB, Sigma-Aldrich, Munich, Germany) in 80% acetonitrile (ACN) and 5% trifluoroacetic acid (TFA). After loading on the TiO_2 column, the sample was washed with loading buffer and 80% ACN, 5% TFA, and eluted with 0.3 M ammonia.

Nano-LC-ESI-MS analysis of enriched UV cross-links. For MS analysis, the sample was dissolved in 50% ACN, 0.1% formic acid (FA) and diluted with 0.1% FA to a final concentration of 10% ACN. The nano-liquid chromatography system (Agilent 1100 series, Agilent Technologies, Böblingen, Germany) was equipped with a C18 trapping column (length ~ 2 cm, inner diameter 150 µm) in-line with a C18 analytical column (length ~ 15 cm, inner diameter 75 µm; both columns packed in-house with C18 AQ 120 Å 5 µm, Dr Maisch GmbH). After injection, the sample was loaded on the trapping column at a flow rate of 10 µL/min in buffer A (0.1% FA v/v) and subsequently eluted and separated on the analytical column with a gradient of 7.5 - 37.5% buffer B (95% ACN, 0.1% FA v/v) within 37 minutes at a flow rate of 300 nL/min.

On-line ESI-MS/MS was performed on a Q-ToF Ultima mass spectrometer (Waters, Manchester, UK). The instrument was operated in data-dependent acquisition mode. Survey scans were recorded for 1 s over the mass range m/z 350–1600. For precursors detected with intensities above 15 counts and a charge state of two to four, a maximum of three consecutive MS/MS scans was triggered.

MS data analysis. MS data analysis for the identification of cross-linked peptides was carried out as described earlier [8] with the exception that only nucleotide combinations present in the RNA sequence (disregarding RNase specificity) were taken into account. In short, the masses of all possible nucleotide combinations were subtracted form the experimental precursor mass of potential cross-links, i.e. spectra not identified as pure peptide or filtered due to small precursor mass or decimal place. The resulting modified masses, together with the unaltered MS/MS fragment

information, were submitted into a database search (MASCOT [10]) to identify the cross-linked peptide moiety. All positive hits were confirmed by close manual inspection of the corresponding experimental MS/MS spectra. For a detailed description of the perl script employed for automated subtraction of calculated oligonucleotide masses as well as the mentioned filtering criteria, please see [8].

References:

1. Niessing D, Hüttelmaier S, Zenklusen D, Singer RH, Burley SK (2004) She2p is a Novel RNA-Binding Protein with a Basic Helical Hairpin Motif. Cell 119: 491-502.
2. Ponchon L, Beauvais G, Nonin-Lecomte S, Dardel F (2009) A generic protocol for the expression and purification of recombinant RNA in Escherichia coli using a tRNA scaffold. Nat Protoc 4: 947-959.
3. Müller M, Richter K, Heuck A, Kremmer E, Buchner J, et al. (2009) Formation of She2p tetramers is required for mRNA binding, mRNP assembly, and localization. RNA 15: 2002-2012.
4. Du TG, Jellbauer S, Müller M, Schmid M, Niessing D, et al. (2008) Nuclear transit of the RNA-binding protein She2p is required for translational control of localized ASH1 mRNA. EMBO Rep 9: 781-787.
5. Adams A, Gottschling DE, Kaiser CA, Stearns T (1997) Methods in Yeast Genetics. Cold Spring Harbor, NY: Cold Spring Harbor Laboratory Press. 1–177 p.
6. Gietz RD, Schiestl RH (1991) Applications of high efficiency lithium acetate transformation of intact yeast cells using single-stranded nucleic acids as carrier. Yeast 7: 253-263.
7. Luo X, Hsiao HH, Bubunenko M, Weber G, Court DL, et al. (2008) Structural and functional analysis of the E. coli NusB-S10 transcription antitermination complex. Mol Cell 32: 791-802.
8. Kramer K, Hummel P, Hsiao H, Luo X, Wahl M, et al. (in press) Mass-Spectrometric analysis of proteins cross-linked to 4-thio-uracil- and 5-bromo-uracil-substituted RNA. Int J Mass Spectrom doi: 10.1016/j.ijms.2010.10.009.
9. Larsen MR, Thingholm TE, Jensen ON, Roepstorff P, Jorgensen TJ (2005) Highly selective enrichment of phosphorylated peptides from peptide mixtures using titanium dioxide microcolumns. Mol Cell Proteomics 4: 873-886.
10. Perkins DN, Pappin DJ, Creasy DM, Cottrell JS (1999) Probability-based protein identification by searching sequence databases using mass spectrometry data. Electrophoresis 20: 3551-3567.
